# Lipid modulation contributes to heat stress adaptation in peanut

**DOI:** 10.3389/fpls.2023.1299371

**Published:** 2023-12-18

**Authors:** William W. Spivey, Sachin Rustgi, Ruth Welti, Mary R. Roth, Mark D. Burow, William C. Bridges, Sruthi Narayanan

**Affiliations:** ^1^ Department of Plant and Environmental Sciences, Clemson University, Clemson, SC, United States; ^2^ Division of Biology, Kansas State University, Manhattan, KS, United States; ^3^ Department of Plant and Soil Sciences, Texas Tech University, Lubbock, TX, United States; ^4^ Texas A&M AgriLife Research and Extension, Lubbock, TX, United States; ^5^ School of Mathematical and Statistical Sciences, Clemson University, Clemson, SC, United States

**Keywords:** peanut, heat stress, leaf lipidome, lipid remodeling, homeoviscous adaptation, triacylglycerol, sterol esters, ox-lipids

## Abstract

At the cellular level, membrane damage is a fundamental cause of yield loss at high temperatures (HT). We report our investigations on a subset of a peanut (*Arachis hypogaea*) recombinant inbred line population, demonstrating that the membrane lipid remodeling occurring at HT is consistent with homeoviscous adaptation to maintain membrane fluidity. A major alteration in the leaf lipidome at HT was the reduction in the unsaturation levels, primarily through reductions of 18:3 fatty acid chains, of the plastidic and extra-plastidic diacyl membrane lipids. In contrast, levels of 18:3-containing triacylglycerols (TGs) increased at HT, consistent with a role for TGs in sequestering fatty acids when membrane lipids undergo remodeling during plant stress. Polyunsaturated acyl chains from membrane diacyl lipids were also sequestered as sterol esters (SEs). The removal of 18:3 chains from the membrane lipids decreased the availability of susceptible molecules for oxidation, thereby minimizing oxidative damage in membranes. Our results suggest that transferring 18:3 chains from membrane diacyl lipids to TGs and SEs is a key feature of lipid remodeling for HT adaptation in peanut. Finally, QTL-seq allowed the identification of a genomic region associated with heat-adaptive lipid remodeling, which would be useful for identifying molecular markers for heat tolerance.

## 1 Introduction

Peanut (*Arachis hypogaea*) is one of the top 10 food crops in the world by area harvested (Food and Agriculture Organization of the United Nations FAOSTAT statistical database, 2022). Peanuts are affordable sources of protein, fatty acids, antioxidants, and vitamins. It offers nutrition to the malnourished and helps address obesity in the over-nourished (Higgs, 2003), which makes it an important crop for addressing hunger and malnutrition in communities of need in the United States and abroad. In the United States alone, an estimated 2,885 thousand metric tons were produced during the 2021-2022 production season, making the United States the fourth largest producer of peanut in the world (United States Department of Agriculture - Foreign Agricultural Service, 2023).

High temperatures associated with climate change threaten peanut production worldwide. Peanut experiences significant decreases in plant growth, pollen performance, peg production, and successful pod formation when the average diurnal temperature exceeds 30°C (Wood, 1968; Cox, 1979; Ketring, 1984; Prasad et al., 1999a; Prasad et al., 1999b; Prasad et al., 2003). Heat-induced yield reduction of peanut is primarily due to the acute sensitivity of two stages of floral development: microsporogenesis and anthesis (Hall, 1992; Gross and Kigel, 1994; Prasad et al., 2001). Consequently, exposure of flower buds and open flowers to temperatures above 33°C for just 12 hours reduces pod set by 40-90%, compared to plants growing at 28°C (Prasad et al., 2001). As the threat of increased temperatures and climate change is being realized, there is a growing need for the development of improved peanut varieties that possess improved heat tolerance (Jagadish et al., 2020; Janni et al., 2020).

Knowledge of the physiological and genetic basis of heat tolerance enables the development of tools for breeding heat-tolerant crop varieties. A fundamental cause of yield loss under heat stress, at the cellular level, is damage to membranes (Ernst et al., 2016). The normal function of the cell membrane depends upon the fluidity of the lipid bilayer, which depends on the composition and properties of its component lipids. Membrane fluidity increases with increases in temperature and unsaturated lipid content. To maintain optimal membrane fluidity under changing temperature conditions, cells regulate membrane lipid composition. This process, termed homeoviscous adaptation, comprises mechanisms that modulate collective membrane properties, i.e., viscosity, lipid packing, thickness, curvature, and phase properties (Seddon and Templer, 1995; Klose et al., 2013; Levental et al., 2016). Mass spectrometry-based lipidomics is currently revealing alterations in the lipid metabolic network during adaptive responses in various crop species including peanut (Larkindale and Huang, 2004; Chen et al., 2006; Schlueter et al., 2007; Li et al., 2015; Narayanan et al., 2016a; Narayanan et al., 2016b; Djanaguiraman et al., 2018; Narayanan et al., 2018; Narayanan et al., 2020; Zhang et al., 2020; Zoong Lwe et al., 2020; Puppala et al., 2023). The influence on homeoviscous adaptation of acyl-chain length, acyl-chain double bond properties (position, number, and configuration), lipid head-group properties (e.g., charge, size, and shape), antifreeze proteins, and divalent metals have been at least partially elucidated. The involvement of other plant lipids, such as triacylglycerols (TG), sterol derivatives, and oxidized lipids (ox-lipids), in the metabolism that supports homeoviscous adaptation is just beginning to be revealed.

Our previous studies utilizing selected peanut genotypes showed evidence of homeoviscous adaptation of membranes in peanut anthers under high temperatures (Zoong Lwe et al., 2020). We found that the lipid unsaturation of membrane phospholipids and levels of 18:3 acyl chains in membrane lipids of peanut anthers decreases at HT (Zoong Lwe et al., 2020). Further, the reduction of 18:3 levels was associated with the reduced expression of a fatty acid desaturase (FAD) enzyme that introduces double bonds in fatty acyl chains [FAD3; converts 18:2 acyl chains to 18:3 in the endoplasmic reticulum (ER)]. Although we confirmed this potential mechanism of homeoviscous adaptation of membranes in peanut anthers, we did not determine the underlying lipid remodeling and mechanistic pathways enabling this phenomenon. In this paper, we report our investigations on a subset of a peanut recombinant inbred line (RIL) population and present the first evidence of peanut leaf lipid remodeling that may serve as a homeoviscous adaptation mechanism to maintain membrane fluidity under heat stress. We are also presenting the results of a DNA sequence analysis that identified a specific genomic region associated with the heat-adaptive lipid remodeling response. An automated direct infusion electrospray ionization triple quadrupole mass spectrometry (ESI-MS/MS) approach was utilized for quantitative profiling of leaf lipids from 52 F_6_ RILs and the parental genotypes grown at optimal temperatures (OT, 30/20°C daytime maximum/nighttime minimum) or high temperatures (HT, 38/28°C) under controlled environmental conditions. We hypothesized that the peanut genotypes would remodel their leaf lipidome as a result of the 14-day heat stress treatment, starting from the initiation of flowering, such that the production of lipid molecules is tailored to ensure membrane functional homeostasis.

## 2 Materials and methods

### 2.1 Plant material

The breeding population used in this study was a population of RILs developed from a cross between ICGS-76 (PI546372) and Tamrun OL02. The population development involved selfing from the F_2_ generation. The genotype ICGS-76 is a Virginia-type breeding line developed by the International Crops Research Institute for the Semi-Arid Tropics (ICRISAT). In previous research, ICGS-76 demonstrated acquired thermo-tolerance (ATT; based on the temperature sensitivity of chlorophyll accumulation), which is likely associated with the induction of heat shock response (Gomez-Selvaraj et al., 2011). By contrast, Tamrun OL02, which is a high-oleic runner-type variety developed by Texas A&M University, had low ATT in the same study (Gomez-Selvaraj et al., 2011). The population used in our study (ICGS-76 x Tamrun OL02) was at the F_6_ generation and consisted of 52 lines. The initial cross was made at the Texas Tech. greenhouse and F_2_ seeds were screened and selected for the high oleic trait by gas chromatography using the fatty acid methyl ester method (Wilson et al., 2017). Breeding lines were evaluated as plant rows beginning with the F_3_ generation. Approximately 180 single plant selections were made from F_5_ breeding lines, and selfed to make the F_6_ generation. Fifty-two of these lines were selected based on seed availability for this study.

### 2.2 Plant husbandry and treatment imposition

Two experiments with the same treatment structure and measurement conditions were conducted under controlled environmental conditions at Clemson University Biosystems Research Complex, Clemson, SC, USA, between February 18, 2022 and November 15, 2022. The seeds were treated with Dynasty PD fungicide (a.i., Azoxystrobin: Methyl (E)-2-{2-[6-(2-cyanophenoxy) pyrimidin-4-yloxy]phenyl}-3-methoxyacrylate, Mefenoxam: (R,S)-2-[(2,6-dimethylphenyl)-methoxyacetylamino]-propionic acid methyl ester, Fludioxonil: 4-(2,2-difluoro-1,3-benzodioxol-4-yl)-1H-pyrrole-3-carbonitrile; Syngenta, Basel, Switzerland) at the rate of 21.8 g kg^-1^ in accordance with the product label prior to sowing. Each pot was amended with the systemic insecticide Marathon (a.i., Imidacloprid: 1-[(6-Chloro-3-pyridinyl)methyl]-N-nitro-2-imidazolidinimine; OHP Inc., Mainland, PA, USA), at the rate of 2.7 g per pot and a controlled-release fertilizer, Osmocote 18-6-12 N:P_2_O_5_:K_2_O (ICL Specialty Fertilizers, Dublin, OH, USA), at the rate of 17 g per pot before sowing. Initially, two seeds of each genotype were sown in 1.37-L pots containing Metro-Mix 830 potting mix (Sun Grow, Anderson, SC, USA) and were later thinned to one plant per pot. All plants were grown in a greenhouse where the daytime and nighttime air temperatures were set at 30 and 20°C, respectively. The daytime temperature was held at 30 ± 1°C and the nighttime temperature at 20 ± 3°C for at least eight consecutive hours (**Figure S1**). The photosynthetic photon flux density, including wavelengths of 400 to 700 nm, measured at the plant canopy height between 11:00 – 13:00 h averaged 1,200 μmol m^-2^ s^-1^ in the greenhouse. The average relative humidity observed for the duration of the experiment was 69%. Plants were watered daily using an automated drip irrigation system. A quick-release fertilizer 15-5-15 N:P_2_O_5_:K_2_O (ICL Group Ltd., Summerville, SC, USA) was incorporated into the irrigation schedule for fertigation every 14 d. Periodic application of pesticides to control plant pests was conducted throughout the duration of the study.

Pots were arranged in the greenhouse using a randomized complete block experimental design with a two-factor factorial treatment design. The treatment factors were genotype and temperature. The experiment was conducted twice to have two runs. Each experimental run consisted of three blocks (replications) and each block included two pots of each of the 54 genotypes. Out of the two pots per genotype in each block, one pot was randomly assigned to OT and the other to HT.

All plants were maintained in the greenhouse under OT conditions (30/20°C) until the onset of flowering (R1, Boote, 1982). Thereafter, two temperature treatments, OT (control, 30/20°C) and HT (heat stress, 38/28°C; Ketring, 1984; Prasad et al., 1999b) were established for 14 d. Plants that received the OT treatment remained in the greenhouse and the plants that received the HT treatment were moved to a growth chamber (BDW160, Conviron, Winnipeg, Canada). The daytime maximum and nighttime minimum temperatures were each held for approximately 12 h in the growth chamber (**Figure S1**). Relative humidity was set at 60% in the growth chamber and the observed values during the temperature treatment period averaged 59%. The quality of temperature control in the growth chamber and the greenhouse is given in **Figure S1**. Air temperature and relative humidity were continuously monitored at 30-min intervals in the growth chamber and greenhouse throughout the experiment using HOBO data loggers (Onset Computer Corporation, Bourne, MA, USA). The photoperiod was 12 h in the growth chamber with the photosynthetic photon flux density measuring 1,200 μmol m^-2^ s^-1^ at the plant canopy level. In the growth chamber, pots were watered daily and kept in trays containing about 1 cm of water throughout the experiment to avoid water stress. The pictures of plants growing in the greenhouse (OT) and growth chamber (HT) are included in **Figure S2**.

### 2.3 Measurement of physiological traits

The chlorophyll index and the efficiency of photosystem II (PSII) were measured on the 13^th^ day of the temperature treatment period. The chlorophyll index, which is a measure of the “greenness” of a leaf, is related to leaf photosynthetic capacity and is a common metric used for assessing the impact of abiotic stress on plants. We measured the leaf chlorophyll index using a self-calibrating chlorophyll meter [Soil Plant Analytical Device (SPAD) 502; Spectrum Technologies, Aurora, IL, USA]. A total of five measurements from the third mature leaf from the apical meristem were averaged to produce a single SPAD value per plant. Chlorophyll fluorescence is a metric that is related to photon capture and photosynthetic rates in plants. We used an OS30p+ modulated fluorometer (Opti-Sciences, Hudson, NH, USA) for measuring chlorophyll fluorescence on the upper left leaflet of the third mature leaf from the apical meristem. The leaflet was dark-adapted for a minimum of 45 min before measuring the minimum fluorescence (F_o_) and the maximum fluorescence (F_m_). The ratio of variable fluorescence (F_v_; the difference between F_m_ and F_o_) to F_m_ represents the maximum quantum yield of PSII and is a metric that indicates the photochemical efficiency of PSII.

### 2.4 Lipid extraction

We collected leaf tissue from a single leaflet of the third mature leaf from the apical meristem between 11:00 and 14:00 h on the 14^th^ day of the temperature treatment for the extraction of lipids. We followed the protocol of Shiva et al. (2018) for lipid extraction. Isopropanol containing 0.01% butylated hydroxytoluene (400 μL) in a 4.0 mL borosilicate glass vial with a PTFE-lined solid cap (Chemglass Life Sciences LLC., Vineland, NJ, USA) was heated to 75°C. Using a hole puncher, we collected five leaf disks from each plant into the hot isopropanol solution and heated the sample for an additional 15 min to deactivate the lipolytic enzymes in the leaf tissue. The vials with leaf disks were allowed to cool to room temperature, and 1.2 mL of chloroform/methanol/water (30/41.5/3.5; v/v/v) were added to each vial. The vials were then placed on an orbital shaker to continuously agitate at 100 rpm for 24 h. After the 24-h shaking period, the lipid extract was transferred to a 2.0-mL borosilicate glass vial with a PTFE-lined solid cap (DWK Life Sciences LLC., Millville, NJ, USA) using a gas-tight syringe (Hamilton, Reno, NV, USA); rinsing the syringe three times with chloroform between samples. The solvent containing the lipid extract was evaporated using an N-EVAP 112 nitrogen evaporator (Organomation Associates, Inc., Berlin, MA, USA), and the lipid extracts were stored at -80°C until shipping to Kansas Lipidomic Research Center (KLRC) for profiling. Vials containing lipid extract were shipped overnight to KLRC on dry ice. The extracted leaf tissue was dried in an oven at 105°C for 24 h, cooled, and weighed using a ME104TE analytical balance (readability of 0.0001 g; Mettler Toledo, Columbus, OH, USA) to express the lipid content on a dry weight basis.

### 2.5 Lipid profiling using an electrospray ionization tandem mass spectrometry

Lipid profiling was carried out using an automated electrospray ionization-tandem mass spectrometry (ESI–MS/MS) approach, as previously described (Song et al., 2020). Direct infusion multiple reaction monitoring was carried out on a triple quadrupole instrument (Sciex 6500+, Sciex Corporation, Framingham, MA, USA) using the parameters shown in **Table S1** and the internal standards indicated in Song et al. (2020) (information about the analysis of the internal standards are also given in **Table S2**). Lipid species in each head-group class were quantified in comparison to the internal standards, as previously described, using the “Average” method for comparison of the analytes with the internal standards (Song et al., 2020). Identical samples of a quality-control pool, prepared by combining aliquots from each lipid sample, were analyzed recurrently among the experimental samples; the resulting data were used to calculate the coefficient of variation of each analyte. The lipid values were calculated as normalized mass spectral signal per milligram of leaf dry weight (**Table S3**), where a value of 1 is the intensity of 1 nmol of internal standard. Lipid analytes for which (1) the amount (normalized mass spectral signal) per milligrams of leaf dry weight was less than the limit of detection (0.00005 nmol) or (2) the coefficient of variation (standard deviation divided by the mean of the amount of the analyte in the quality-control samples) was greater than 0.3 were removed from the data set in order to maintain data quality. The lipid classification, nomenclature, and shorthand notation followed the latest updates by Liebisch et al. (2020).

### 2.6 Product ion analysis of triacylglycerols

In order to determine all three acyl chains of ambiguous TG species, we conducted product ion analysis using a XEVO-TQS triple quadrupole mass spectrometer (Waters Corporation, Milford, MA, USA). TG molecular species were identified by examining the *m/z* of each of the fragments, produced by neutral loss of fatty acid + NH_3_, when the ammonium adduct (i.e., [M + NH_4_]^+^) of the TG was subject to a product ion scan in positive mode. Results are shown in **Table S4**.

### 2.7 Lipid unsaturation index

The unsaturation index indicates the number of double bonds in a lipid and was calculated in this study using a previously published formula (Narayanan et al., 2016a; Narayanan et al., 2018). The unsaturation index for a lipid molecular species refers to the average number of double bonds per acyl chain, which was calculated as the number of double bonds in the lipid molecular species divided by the number of acyl chains. The unsaturation index of a lipid head-group class was calculated as:


$$\frac{\sum^{}\text{(unsaturation indices of individual lipid molecular species in the class }\times \text{ amount of each species)}}{\sum^{}\text{amount of all lipid molecular species in the class}}$$

### 2.8 Statistical analysis

We conducted an analysis of variance (ANOVA) and estimated the least-squares means and standard errors using the GLIMMIX procedure in SAS (v9.4, SAS Institute Inc.). In the statistical analysis, temperature treatment, genotype, and the temperature treatment x genotype interaction were considered as fixed effects and replication as a random effect. The effect of experimental run and the interactions involving run were not significant on most traits, and data from the two experimental runs were pooled together for the analysis. The ANOVA results are presented in **Table S5**. Separation of least-squares means was conducted based on Fisher’s least significant difference (LSD) test (α = 0.05) using the LSMEANS option in the GLIMMIX procedure. The R Statistical Software (v4.2.1; R Core Team, 2021) was used to conduct the principal component analysis and clustering analysis. Figures were created using JMP Pro16 (v16.1.0; SAS Institute Inc.) and the R packages ggplot2 (v3.4.2; Wickham, 2016), factoextra (v1.0.7; Kassambara and Mundt, 2020), ggpubr (v.0.6.0; Kassambara, 2023), and pheatmap (v.1.0.12; Kolde, 2019).

### 2.9 DNA extraction and sequencing, sequence analysis, and QTL mapping

Leaf tissue from all 54 genotypes (52 RILs and two parental genotypes) was collected and used for DNA extraction and genomic sequencing. The tissue was collected and frozen at -80°C until processing. DNA extraction was conducted using Zymo Research Quick-DNA Plant/Seed Miniprep kit (Zymo Research, Irvine, CA, cat. D6020) following the manufacturer’s recommendations.

All 54 genotypes were sequenced on the NovaSeq 6000 platform with S4 flow cells at the Hudson Alpha Institute for Biotechnology, Huntsville, AL, USA. The paired-end sequences of ~150bp were obtained for each of the 52 RILs and the parental genotypes. Two RILs (#5 and #24) resulted in low-depth sequences (1,714,572 and 1,643,109 reads) and were excluded from further analysis. The rest of the RILs produced a range of 4,667,080 (RIL W.01) to 64,428,457 (RIL W.04) reads (**Table S9**). After filtering, the sequences were aligned to the peanut reference genome of cv. Tifrunner. Sequenced lines were analyzed using the Khufu pipeline (https://www.hudsonalpha.org/khufudata/, accessed on 22 August 2023). A freely accessible shiny app (https://w-korani.shinyapps.io/khufu_var2/) was used to select from the Khufu processed data. This software reduces the background noise by filtering low-depth loci and loci with low minor allele frequency (Korani et al., 2021).

The level of polymorphism across subgenomes and chromosomes was determined and compared with the expected number of polymorphisms determined per chromosome, assuming an even distribution of SNPs across chromosomes and genomes. The expected number of markers per chromosome was determined by dividing the total number of markers per subgenome by the sub-genome size in Mb and multiplying the number by chromosome size in Mb (Bertioli et al., 2019). Similarly, at the chromosome level, the expected number of markers in 10 Mb bins throughout the chromosome length was determined by dividing the total number of the markers by the chromosome size in Mb and multiplying the number by ten. Diversity among RILs and parental genotypes was studied using the marker data. Ward’s hierarchical clustering was used to cluster the RILs and parental genotypes based on the genotypic data which was presented on a dendrogram produced using the JMP Statistical Software package.

## 3 Results

### 3.1 Heat impact on the peanut population based on physiological traits

**Figure 1:**
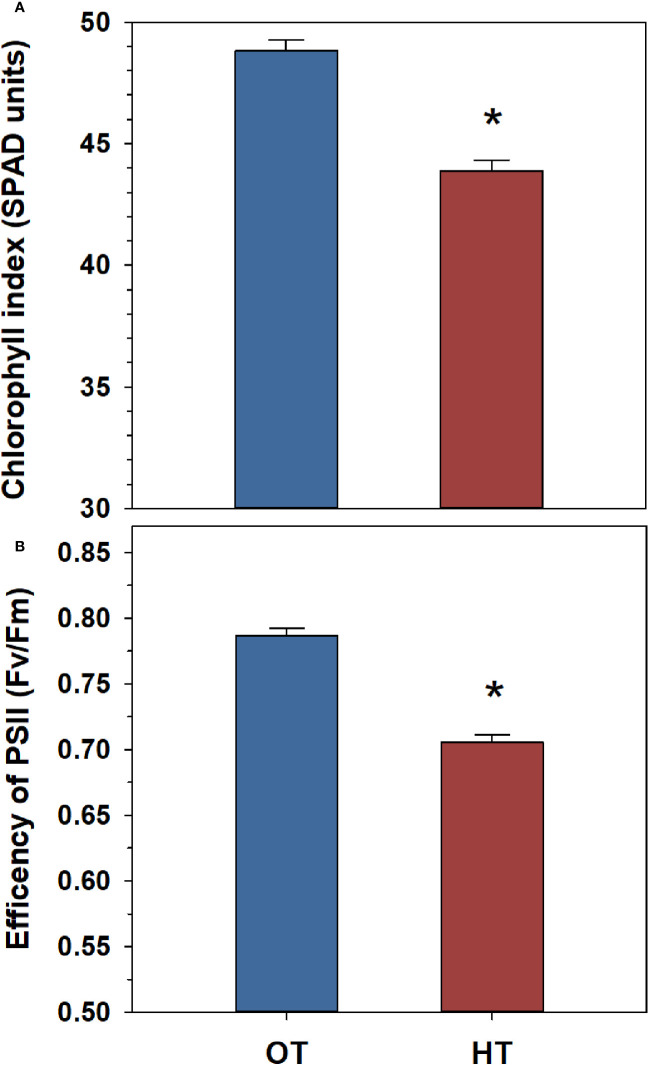
Temperature effects on chlorophyll index **(A)** and the efficiency of photosystem II (PSII) **(B)** in a peanut population of 54 genotypes (52 recombinant inbred lines and the parental genotypes, ICGS-76 and Tamrun OL02). Values shown are the least-squares means. Error bars represent the standard errors of the least-squares means of 324 observations (2 experimental runs x 54 genotypes x 3 replications). An asterisk (*) above the bars indicates a significant difference between optimal temperature (OT, 30/20°C) and heat stress (HT, 38/28°C) at α = 0.05 according to the Fisher’s least significant difference (LSD) test.

Heat stress had significant impacts on the physiological traits measured in the peanut population (**Table S5**). Overall, we observed a significant decrease in the chlorophyll index and the efficiency of PSII under heat stress (**Figure 1**). The chlorophyll index and the efficiency of PSII each decreased by 11% under heat stress (**Figure 1**).

### 3.2 Composition of the peanut leaf lipidome

**Figure 2:**
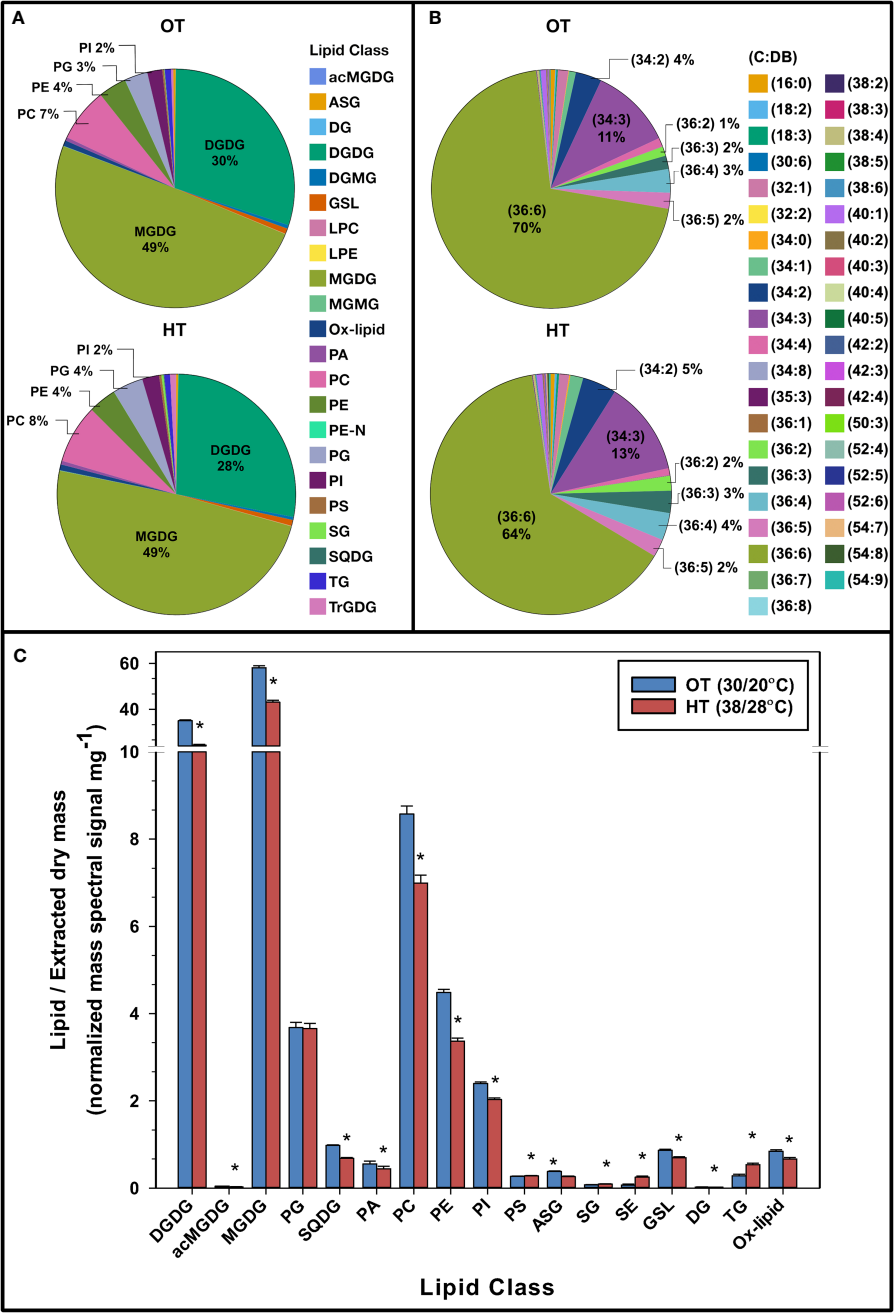
Pie charts representing the composition of various lipid headgroup classes **(A)**, lipid molecular species (C:DB- total acyl carbons: total double bonds) **(B)**, and the total amount of lipids in various head group classes **(C)** in peanut leaves under optimal temperature (OT) and high temperature (HT). The values shown in panel **(C)** are the least-squares means. Error bars in panel **(C)** represent the standard errors of the least-squares means of 324 observations (2 experimental runs x 54 genotypes x 3 replications). A break on the y-axis in panel **(C)** indicates a change in scale. An asterisk (*) above the bars indicates a significant difference between OT and HT at α = 0.05 according to the Fisher’s least significant difference (LSD) test. acMGDG, acylated MGDG; ASG, acylated sterol glycoside; DG, diacylglycerol; DGDG, digalactosyldiacylglycerol; DGMG, digalactosylmonoacylglycerol; GSL, glycosphingolipid; LPC, lysophosphatidylcholine; LPE, lysophosphatidylethanolamine; MGDG, monogalactosyldiacylglycerol; MGMG, monogalactosylmonoacylglycerol; ox-lipid, oxidized lipid; PA, phosphatidic acid; PC, phosphatidylcholine; PE, phosphatidylethanolamine; PE-N, N-acyl PE; PG, phosphatidylglycerol; PI, phosphatidylinositol; PS, phosphatidylserine; SE, sterol ester; SG, sterol glycoside; SQDG, sulfoquinovosyldiacylglycerol; TG, triacylglycerol; TrGDG, trigalactosyldiacylglycerol.

Leaf samples, for lipid profiling using an ESI–MS/MS approach, were collected from the peanut plants on the last day of the two-week temperature treatment. The quantities of 192 lipid species detected in the peanut leaves are shown in **Table S3**. Under OT and HT, 80% of the mass spectral signal from the peanut leaf lipidome was from MGDG (49%) and DGDG (28-30%) (**Figure 2A**). Other major components of the lipidome were PC (7-8% of mass spectral signal), PE (4%), PG (3-4%), and PI (2%) (**Figure 2A**). The total amount of DGDG, MGDG, SQDG, PA, PC, PE, PI, DG, ox-lipids, ASG, and GSL decreased under HT, whereas the total amount of SE, SG, and TG increased (**Figure 2C**).

When analyzing the lipid molecular species composition of structural glycerolipids in the peanut plants’ leaf lipidome, 36:6 species (containing two 18:3 acyl chains) were the most prominent under OT (70%) and HT (64%) (**Figure 2B**). The second most abundant lipid species was 34:3 (11% under OT and 13% under HT). Other abundant species were 34:2 (4% under OT and 5% under HT), 36:4 (3% under OT and 4% under HT), 36:5 (2% under OT and HT), and 36:3 (2% under OT and 3% under HT). All of the above species contained one or two C18 or C16 chains, which are the most common acyl chains in higher plants.

### 3.3 Differences in membrane diacyl lipids in relation to temperature treatment

Principal component analysis indicates that certain lipids distinguish the heat treatment from the control treatment (OT) indicating their significant involvement in plant response to heat stress (**Figures S3**, **S4**). Membrane diacyl lipids that were distinctly different in plants exposed to OT vs HT were the DGDG, MGDG, PG, and SQDG plastidic lipids (**Figure S3**) and PC, PE, PI, and PS extra-plastidic lipids (**Figure S4**). In each case, principal component analysis of the molecular species within the class separated the OT and HT samples. Clusters formed in the analyses of DGDG, MGDG, and PG lipids did not include any overlap (i.e., no data point corresponding to OT fell in the HT cluster or vice versa) when shaded with a 95% confidence ellipse. The other plastidic lipid class, SQDG, and all the extra plastidic lipid classes PE, PC, PI, and PS also separated the temperature treatments into clusters, but there were overlaps between the clusters (i.e., some data points corresponding to OT fell in the HT cluster or vice versa) (**Figures S3**, **S4**).

### 3.4 Remodeling of the peanut leaf lipidome under heat stress

#### 3.4.1 Unsaturation levels of plastidic and extra-plastidic membrane diacyl lipids decreased under heat stress

**Figure 3:**
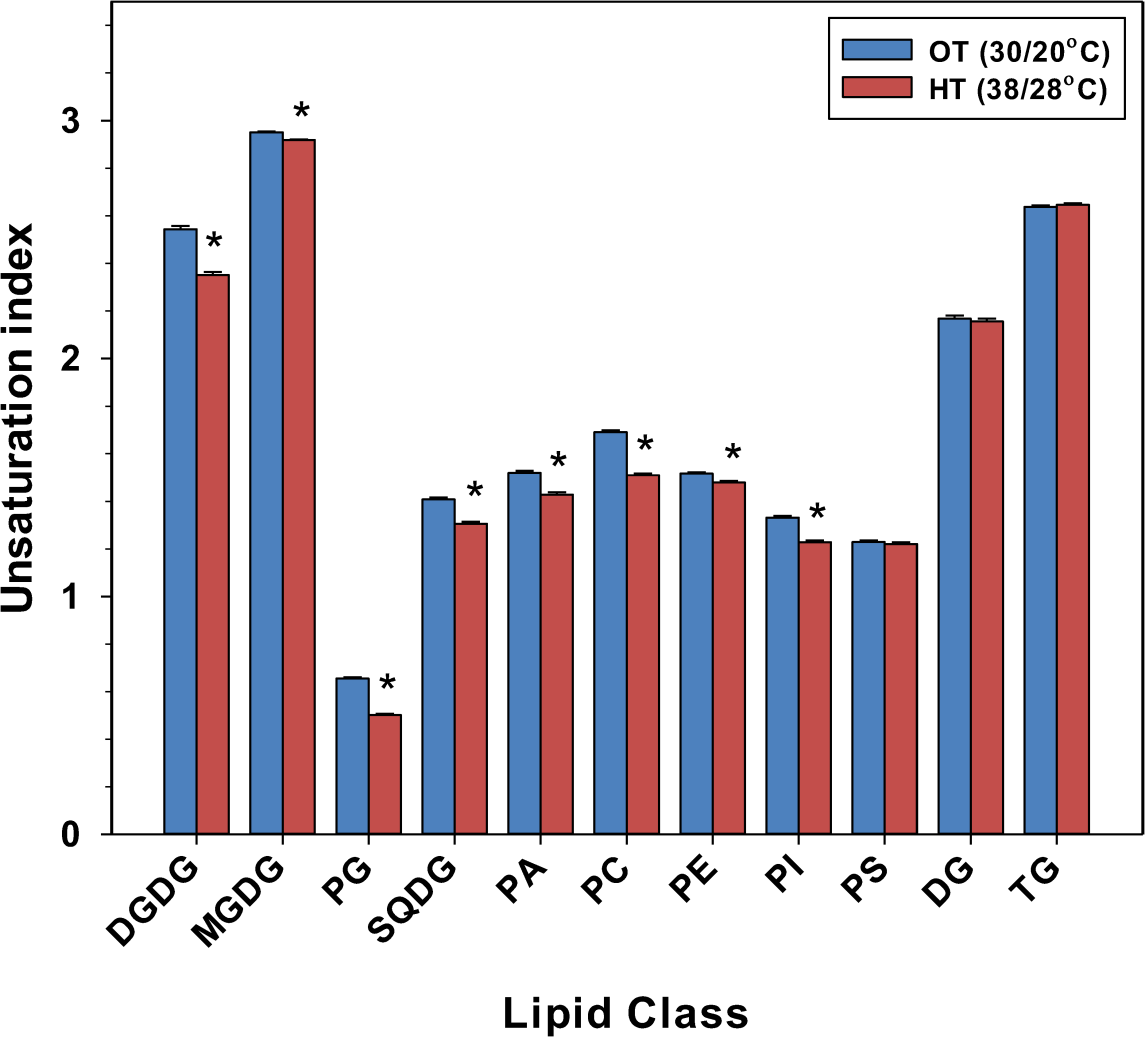
Temperature effects on the unsaturation index of various lipid head-group classes in peanut leaves. The unsaturation index for a lipid molecular species was calculated as the average number of double bonds per acyl chain, which is the number of double bonds in the lipid molecular species divided by the number of acyl chains. The unsaturation index of a lipid head-group class was calculated as: sum of (unsaturation indices of individual lipid molecular species in the class x amount of each species) divided by the sum of the amount of all lipid molecular species in the class. The values shown are the least-squares means. Error bars represent the standard errors of the least-squares means of 324 observations (2 experimental runs x 54 genotypes x 3 replications). An asterisk (*) above the bars indicates a significant difference between optimal temperature (OT) and high temperature (HT) at α = 0.05 according to the Fisher’s least significant difference (LSD) test. DG, diacylglycerol; DGDG, digalactosyldiacylglycerol; MGDG, monogalactosyldiacylglycerol; PA, phosphatidic acid; PC, phosphatidylcholine; PE, phosphatidylethanolamine; PG, phosphatidylglycerol; PI, phosphatidylinositol; PS, phosphatidylserine; SQDG, sulfoquinovosyldiacylglycerol; TG, triacylglycerol.

In peanut leaves, the amounts of more unsaturated plastidic and extra-plastidic diacyl lipid species decreased and the amounts of less unsaturated plastidic and extra-plastidic diacyl lipid species increased under HT, as compared to OT (**Figures S5**, **S6**). For example, 36:6- (which is a di-18:3 combination) DGDG, MGDG, PG, SQDG, PC, PE, PI, and PA, decreased under HT. Specifically, most lipid species that have at least one 18:3 acyl chain decreased under HT (**Table S6**). On the other hand, most lipid species containing saturated acyl chains (i.e., 16:0 or 18:0) or monounsaturated acyl chains (i.e., 18:1), and no 18:3 acyl chain increased under HT (**Table S6**). By decreasing the levels of 18:3 acyl chains and increasing the levels of saturated or monounsaturated acyl chains (16:0, 18:0, and/or 18:1) (**Figures S5**, **S6**; **Table S6**), peanut plants decreased the unsaturation indices of most membrane diacyl lipid classes, DGDG, MGDG, PG, SQDG, PC, PE, PI, and PA (**Figure 3**).

To determine the fold changes of fatty acids of various lipid classes in response to HT, the amount of each fatty acid in each lipid class was summed, based on the lipid molecular species, and compared between OT and HT (**Table 1**). This analysis showed that 18:3 levels experienced a fold decrease across all membrane diacyl lipid classes except PS, and a fold increase in TG and ASG under HT. On the other hand, saturated fatty acid 18:0 and monounsaturated fatty acid 18:1 showed fold increases in many membrane diacyl lipid classes.

**Table 1:** Fatty acyl chain (total number of C: total number of double bonds) fold change under high temperatures in the major lipid classes.

		Acyl Chains															
		16:0	16:1	17:0	18:0	18:1	18:2	18:3	20:0	20:1	20:2	20:3	22:0	22:1	22:2	24:0	24:1
Lipid Class	DGDG	**0.90**	**0.61**		**1.75**	0.99	0.97	**0.64**		**0.91**	**0.66**	**0.73**					
	MGDG	**1.35**	**0.65**	**1.78**	**1.94**	**0.89**	**1.32**	**0.73**		**0.66**	**0.83**	**0.83**					
	PG	**1.16**	**0.64**		**1.50**	**1.44**	**0.73**	**0.73**									
	PA	**0.82**	**0.75**		**0.88**	1.06	0.90	**0.60**									
	PC	**0.95**	**0.71**		**0.94**	**1.38**	**0.85**	**0.60**	**0.78**	**0.60**			**0.74**	**0.65**	**0.70**		
	PE	**0.71**	**0.59**		**0.79**	**0.83**	**0.88**	**0.53**	**0.86**		**0.59**	**0.51**	**0.77**			**0.77**	**0.73**
	PI	**0.89**	**0.92**		**0.89**	**0.83**	0.97	0.67									
	PS	**0.67**	**0.55**				**1.03**	**1.05**					**1.35**			**0.82**	
	DG	**0.82**					1.03	**0.82**									
	ASG	**0.88**					**0.58**	**0.46**									
	TG	0.96					**2.10**	**1.93**									
	SE						**5.42**	**2.52**									

Lipid molecular species for which the fatty acyl composition is unambiguous were used for calculations. Fold change value for each fatty acyl chain in each lipid class was calculated as [the sum of (amount of lipid species times the number of occurrences of the fatty acid in that lipid species) at high temperature]/[the sum of (amount of lipid species times the number of occurrences of the fatty acid in that lipid species) at optimal temperature] (Zoong Lwe et al., 2020). Red shades represent increases and blue shades represent decreases in fold change. Bold number formatting indicates a significant difference between the acyl chain value between the temperature treatments at α = 0.05 according to Fisher’s least significant difference (LSD) test. The optimal temperature was 30/20°C and high temperature was 38/28°C. ASG, acylated sterol glycoside; DG, diacylglycerols; DGDG, digalactosyldiacylglycerol; MGDG, monogalactosyldiacylglycerol; PA, phosphatidic acid; PC, phosphatidylcholine; PE, phosphatidylethanolamine; PG, phosphatidylglycerol; PI, phosphatidylinositol; PS, phosphatidylserine; TG, triacylglycerol; SE, sterol ester.

#### 3.4.2 18:3-containing triacylglycerols increased under heat stress

**Figure 4:**
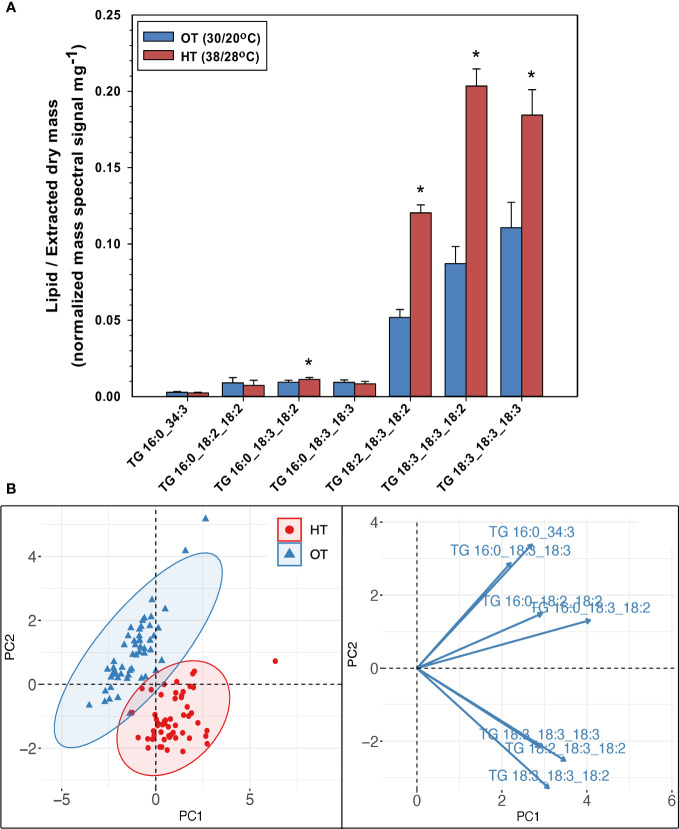
Changes in the levels of triacylglycerol (TG) species in peanut leaves in response to temperature treatments **(A)**, and principal component analysis (PCA) biplot demonstrating the differentiation of the two temperature treatments: optimal temperature (OT) and high temperature (HT) by the TG species **(B)**. The values shown in panel **(A)** are the least-squares means. Error bars in panel **(A)** represent the standard errors of the least-squares means of 324 observations (2 experimental runs x 54 genotypes x 3 replications). An asterisk (*) above the bars in panel **(A)** indicates a significant difference between OT and HT at α = 0.05 according to the Fisher’s least significant difference (LSD) test. Lipid molecular species are identified as total acyl carbons:total double bonds.

While the amounts of plastidic and extra-plastidic membrane lipid species that contained 18:3 acyl chains at high temperatures decreased in peanut leaves, the levels of TG species that contained one, two, or three 18:3-acyl chains (16:0_18:3_18:2, 18:2_18:3_18:2, 18:3_18:3_18:2, 18:3_18:3_18:3 TGs) increased under HT (**Figure 4A**). TG species that contained two or more 18:3 acyl chains increased by 95% under HT. The total amount of TGs also increased by 91% under HT (**Figure 2C**). Further, supporting their involvement in heat stress adaptation, TGs also separated the temperature treatments into distinct clusters (**Figure 4B**).

#### 3.4.3 Decreased unsaturation levels in plastidic and extraplastidic diacyl membrane lipids were accompanied by decreased oxidation of polyunsaturated fatty acids

Oxidized fatty acyl chains are designated by indicating the number of carbons, number of double bond equivalents, and number of oxygens in addition to the carbonyl oxygen (Buseman et al., 2006; Ibrahim et al., 2011; Vu et al., 2012). For example, DGDG 16:0_18:3;O indicates the lipid head group as DGDG, the non-oxidized acyl chain as 16:0, and the oxidized acyl chain as 18:3;O (18 carbons, three C-C double bonds, and one oxygen atom in addition to the carbonyl oxygen).

**Figure 5:**
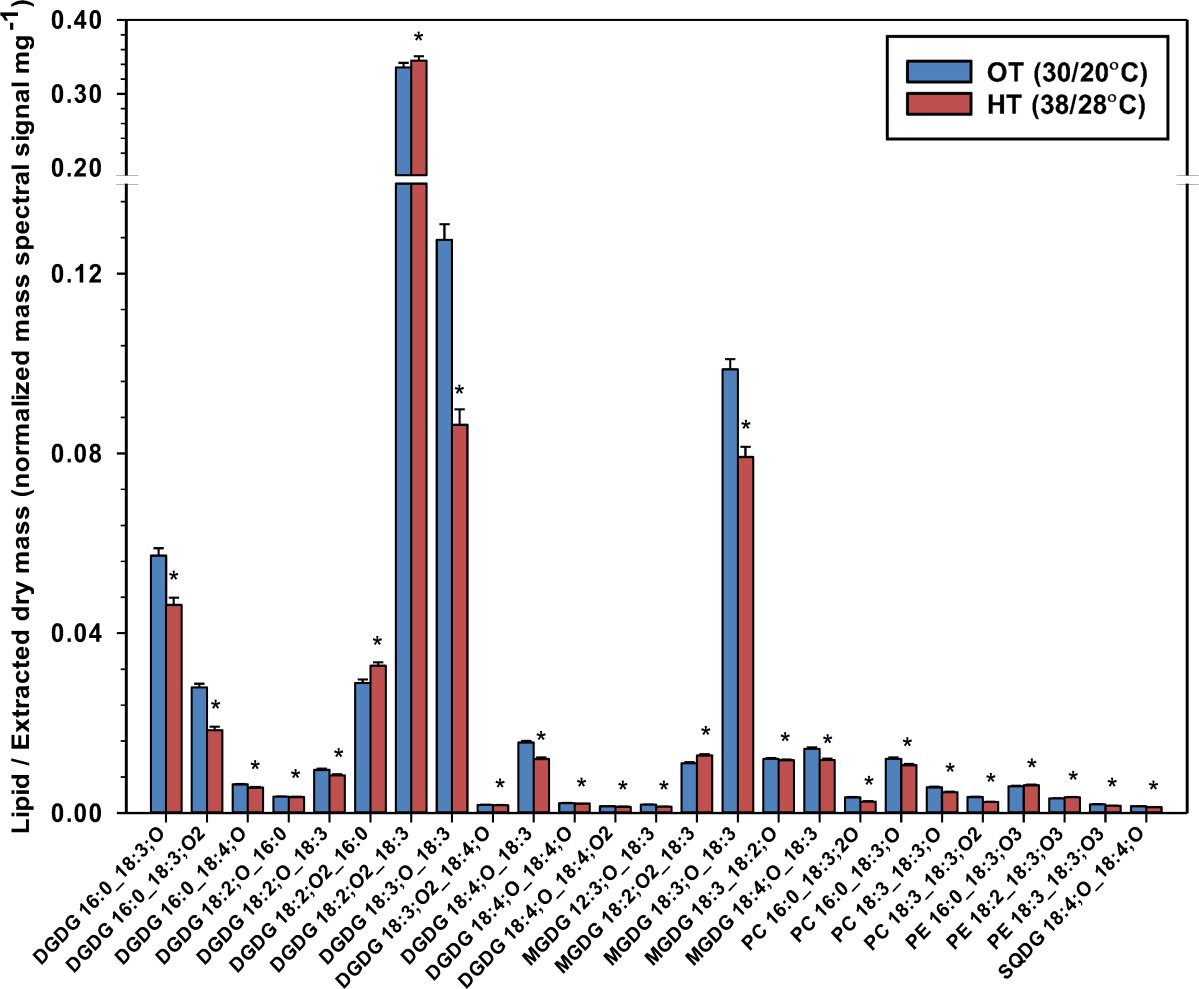
Temperature effects on lipids that contain oxidized fatty acids (ox-lipids) in peanut leaves. Ox-lipids are designated by indicating the number of carbons, number of double bond equivalents, and number of oxygens in addition to the carbonyl oxygen. The values shown are the least-squares means. Error bars represent the standard errors of the least-squares means of 324 observations (2 experimental runs x 54 genotypes x 3 replications). An asterisk (*) above the bars indicates a significant difference between optimal temperature (OT) and high temperature (HT) at α = 0.05 according to the Fisher’s least significant difference (LSD) test. DGDG, digalactosyldiacylglycerol; MGDG, monogalactosyldiacylglycerol; PC, phosphatidylcholine; PE, phosphatidylethanolamine; PG, phosphatidylglycerol; SQDG, sulfoquinovosyldiacylglycerol.

In our study, oxidized fatty acyl chains were found in DGDG (12 species), MGDG (5 species), SQDG (1 species), PC (4 species), and PE (3 species). Oxidized lipids (ox-lipids) contained oxidized polyunsaturated fatty acid (PUFA) chains. Most ox-lipids, perhaps unexpectedly, significantly decreased under HT (**Figure 5**), likely due to the removal of the substrate for their formation, 18:3, from the membrane. In *Arabidopsis*, 18:3 and 16:3 acyl chains in galactolipids are commonly oxidized under HT giving rise to 18:4;O (oxophytodienoic acid, OPDA) and its 16-carbon analog, 16:4;O (dinor-OPDA; dnOPDA] (Shiva et al., 2020), as well as many other oxidized molecular species. Note that in the ox-lipids, the number of double bonds includes all double bond equivalents, which may include rings or ketones, as is the case in OPDA. While none of the ox-lipids have been characterized beyond the formula level, the oxidized 18:3;O and 18:2;O chains are likely hydroxy fatty acids. The 18:4;O chain was found attached only to the plastidic lipids (DGDG, MGDG, and SQDG), which may suggest that it represents OPDA, as the conversion of 18:3 to OPDA (i.e., 18:4;O) takes place in the chloroplast (Nilsson et al., 2012).

#### 3.4.4 Sterol esters and sterol glycosides accumulated under heat stress

**Figure 6:**
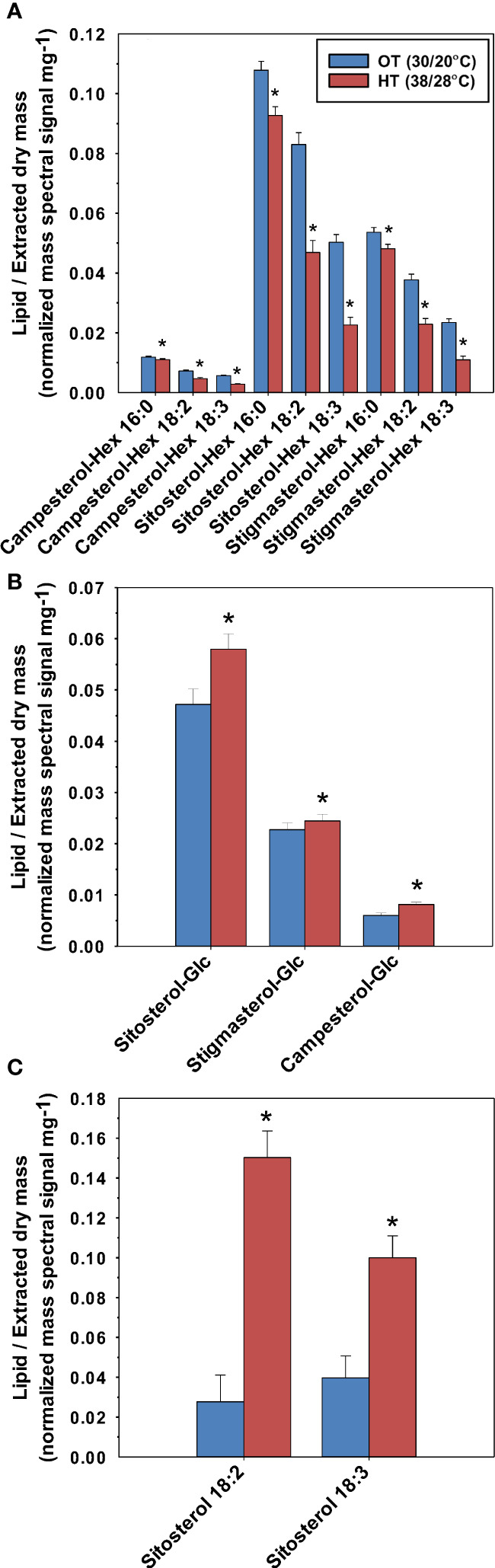
Effects of temperature on the molecular species of acylated sterol glycosides (ASG) **(A)**, sterol glycosides (SG) **(B)**, and sterol esters (SE) **(C)** in peanut leaves. OT, optimal temperature; HT, high temperature. The values shown are the least-squares means. Error bars represent the standard errors of the least-squares means of 324 observations (2 experimental runs x 54 genotypes x 3 replications). An asterisk (*) above the bars indicates a significant difference between OT and HT at α = 0.05 according to the Fisher’s least significant difference (LSD) test.

All three forms of conjugated phytosterols (SE, SG, and ASG) were detected in the peanut leaves (**Figure 6**). There were campesterol, sitosterol, and stigmasterol derivatives. Linoleic acid (18:2) and linolenic acid (18:3) were esterified to sitosterol, forming the measured SEs: sitosterol 18:2 and sitosterol 18:3 (**Figure 6C**). Both SEs increased under HT: 152% increase for sitosterol 18:3 and 442% increase for sitosterol 18:2. Additionally, all three SG species increased under HT; Sitosterol-Glc by 23%, Stigmasterol-Glc by 8%, and Campesterol-Glc by 36%. Palmitic acid (16:0), linolenic acid, and linoleic acid were esterified to the SGs, forming nine ASG species, all of which decreased under HT.

### 3.5 Lipids undergoing coordinated metabolism under heat stress

**Figure 7:**
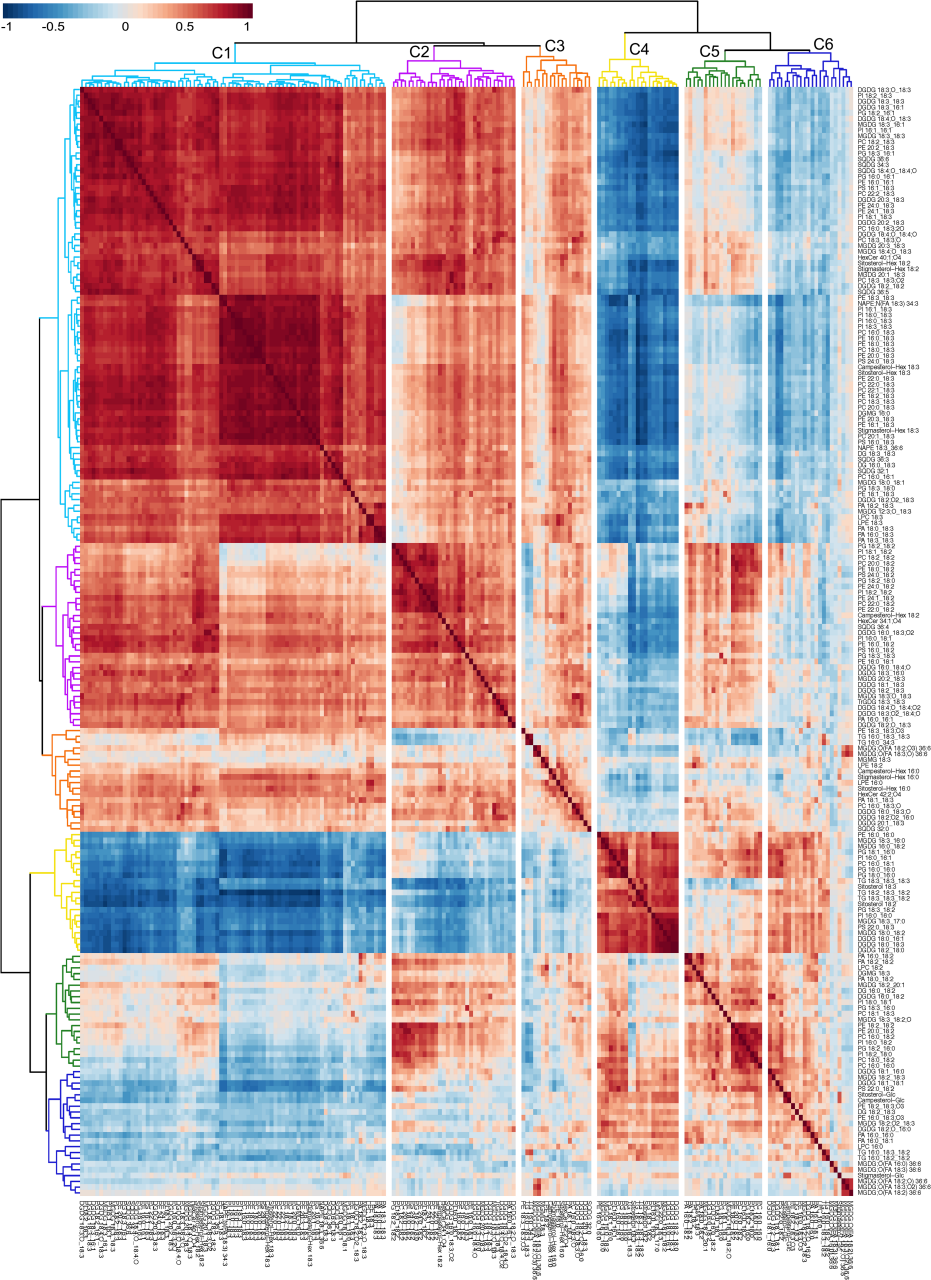
Dendrogram and heat map demonstrating co-occurring lipid groups in peanut leaves. All 192 lipid species were clustered using a complete-linkage hierarchical clustering approach based on Spearman’s correlation coefficient, ρ. The value of ρ ranges from -1 (perfect negative correlation) to 1 (perfect positive correlation), with ρ = 0 indicating no correlation. Red color on the heat map indicates positive values while blue color indicates negative values of ρ. Six clusters of lipids (C-1 through C-6) are color-coded on the figure. Lipids included in the clusters are listed in Supporting Information **Table S8**. ASG, acylated sterol glycoside; DG, diacylglycerol; DGDG, digalactosyldiacylglycerol; DGMG, digalactosylmonoacylglycerol; GSL, glycosphingolipid; LPC, lysophosphatidylcholine; LPE, lysophosphatidylethanolamine; MGDG, monogalactosyldiacylglycerol; MGMG, monogalactosylmonoacylglycerol; ox-lipid, oxidized lipid; PA, phosphatidic acid; PC, phosphatidylcholine; PE, phosphatidylethanolamine; PE-N, N-acyl PE; PG, phosphatidylglycerol; PI, phosphatidylinositol; PS, phosphatidylserine; SE, sterol ester; SG, sterol glycoside; SQDG, sulfoquinovosyldiacylglycerol; TG, triacylglycerol; TrGDG, trigalactosyldiacylglycerol.

Previous studies have reported the use of a dendrogram to detect lipids experiencing coordinated metabolism based on the analysis of lipid co-occurrence (Vu et al., 2014; Narayanan et al., 2016b; Zoong Lwe et al., 2020). To produce a dendrogram, we calculated the Spearman correlation coefficients (ρ) among lipid analytes across the 54 peanut genotypes under OT and HT (**Table S7**). A dendrogram was then created by matching each lipid analyte with the one to which it was most highly correlated based on Spearman’s correlation coefficient (**Figure 7**). Hierarchical clustering analysis using the complete linkage method based on Euclidean distance classified the 192 detected lipid species into six clusters on the dendrogram. The clusters (**Table S8**) reflect the grouping of lipid species according to the patterns of their accumulation changes under HT. Lipids that contained 18:3 acyl chains across different diacyl membrane lipid classes and that decreased in amounts under HT formed a large cluster (cluster 1) and showed strong positive correlations among themselves, supporting their co-occurrence due to coordinated metabolism. The 18:3-acyl-containing TGs and the SEs, sitosterol 18:2 and sitosterol 18:3, which increased in amounts under HT were included in cluster 4, supporting their co-occurrence. The TGs and SEs also showed strong correlations among themselves, supporting their co-occurrence, and coordinated metabolism. Cluster 1 and cluster 4 exhibited a negative correlation with each other. Co-occurrence was also observed for lipids with oxidized 18:3 acyl chains that decreased in amounts under HT and grouped into clusters 1, 2, and 3. Clusters 1 and 2 exhibited the strongest positive correlations while clusters 4 and 6 had the strongest negative correlations.

### 3.6 Contrasting genotypes in terms of heat-adaptive lipid metabolism

**Figure 8:**
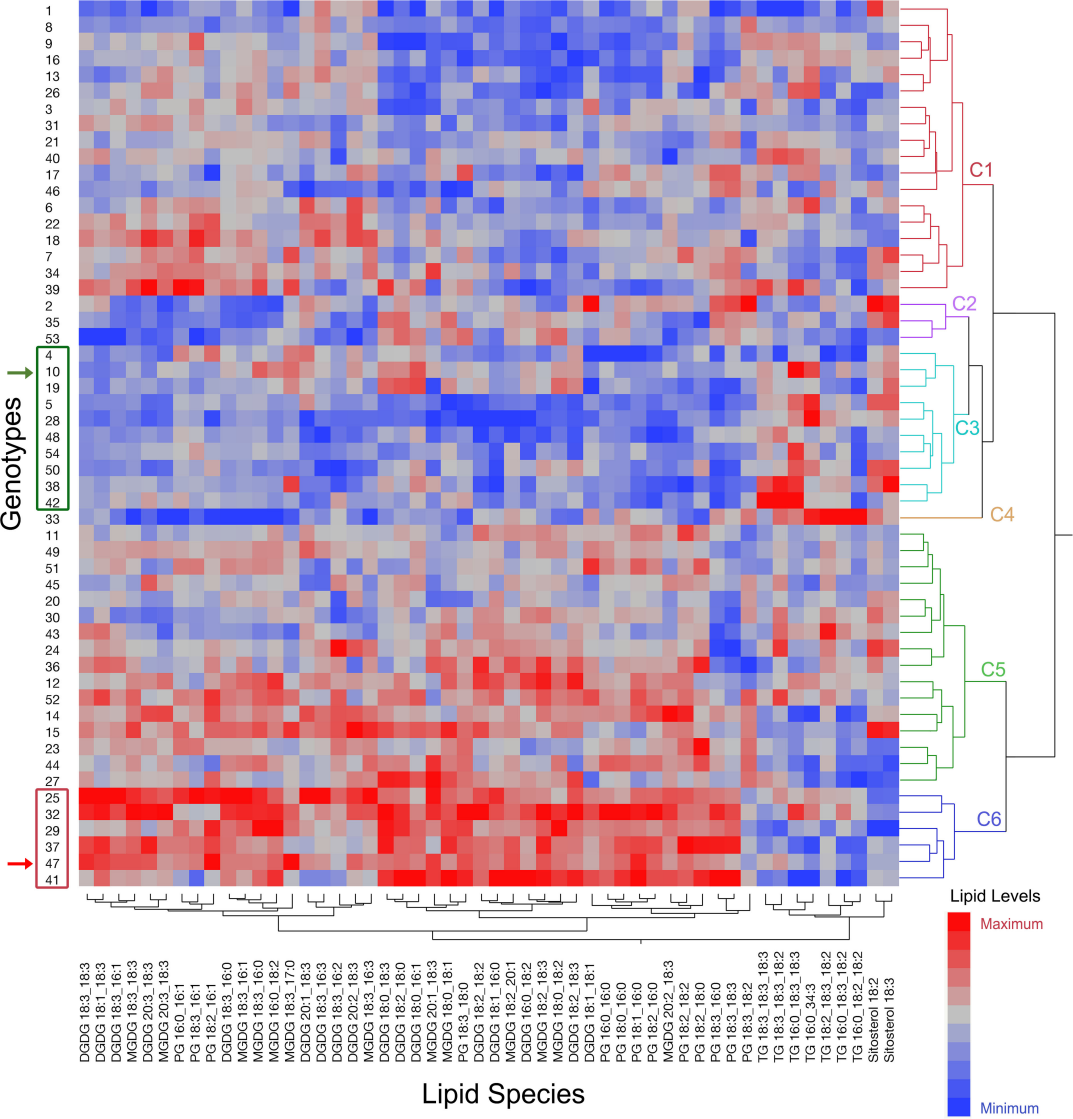
Ward’s two-way hierarchical clustering dendrogram of the 54 genotypes utilizing the levels of DGDG, MGDG, PG, TG, and SE lipid species at high temperature. Genotypes were clustered into six clusters: C1 to C6. Red color on the heat map indicates high values while blue color indicates low values for the amount of the corresponding lipid species given at the bottom of the figure. Genotype 47 is Tamrun OL02 which was the heat susceptible parent (indicated by red arrowhead), and genotype 10 is ICGS76 which was the heat tolerant parent (indicated by green arrowhead). Genotypes included in cluster 3 and cluster 6 which also included either the heat-tolerant parent or the heat-susceptible parent, respectively, are highlighted by green and red rectangles. DGDG, digalactosyldiacylglycerol; MGDG, monogalactosyldiacylglycerol; PG, phosphatidylglycerol; TG, triacylglycerol; SE, sterol ester.

Contrasting genotypes were identified based on three features of the heat-adaptive lipid metabolism explained above; (1) alterations in DGDG, MGDG, and PG lipid classes best distinguished the temperature treatments as indicated by the lack of overlap in their confidence ellipses (α = 0.05) in **Figure S3**, (2) a major heat-adaptive alteration in the peanut leaf lipidome was the reduction in the amount of 18:3 acyl chains in membrane diacyl lipids, and (3) the 18:3 acyl chains expelled from the membrane diacyl lipids were likely sequestered into TG and SE lipid species. Thus, we clustered the genotypes (Ward’s hierarchical clustering using JMP Pro 16 software) based on the levels of DGDG, MGDG, PG, TG, and SE lipid species at HT (**Figure 8**). The 54 peanut genotypes were grouped into six clusters (**Figure 8**). Based on the heat-adaptive lipid metabolism features listed above, the genotypes in cluster 3 which generally displayed the lowest levels of the 18:3 containing DGDG, MGDG, and PG and the highest levels of the 18:3 containing TG and SE were classified as the most heat-tolerant genotypes. Similarly, the genotypes that were grouped into cluster 6 which displayed the highest levels of the 18:3 containing DGDG, MGDG, and PG, and the lowest levels of the 18:3 containing TG and SE were classified as the most heat-susceptible genotypes. The heat-tolerant parent, ICGS-76, was included in cluster 3 with the genotypes identified as the most heat tolerant and the heat-susceptible parent, Tamrun OL02, was included in cluster 6 with the genotypes identified as the most heat-susceptible. This result further supported the identification of the contrasting genotypes. The SNP haplotype of the genotypes in cluster 3 [including the heat-tolerant parental genotype, ICGS76 (W.10) and the RILs showing heat-adaptive lipid metabolism] and cluster 6 [including the heat-susceptible parental genotype, Tamrun OL02 (W.47) and the RILs that exhibited unfavorable heat-responsive lipid metabolism] were compared with that of the reference peanut genotype Tifrunner used for DNA sequencing (Bertioli et al., 2019) (**Figure S7**). Interestingly, the SNP haplotype of the putative heat-tolerant genotypes and the heat-tolerant parental genotype ICGS76 resembled that of Tifrunner, which is also regarded as drought-tolerant (Puppala et al., 2023).

### 3.7 Genetic regulation of heat-adaptive lipid metabolism

The sequence analysis allowed the identification of 13,962 SNPs distributed across the genome with a minimum number of markers (158) mapping to chromosome 8 (A-subgenome) and a maximum number of markers (3,300) mapping to chromosome 19 (B-subgenome). The number of markers on chromosome 8 was significantly less and that on chromosome 19 was significantly greater than the expected values determined using the chromosome sizes (**Figure S8**). The distribution of markers across the chromosome length was also studied (**Figure S9**). No general trends were observed between homoeologous chromosomes, within subgenomes, or at the genome-scale. Peaks of increased diversity were observed at different chromosomal locations, such as telomeric, sub-telomeric, and centromeric regions on either chromosome arms. An exceptionally high level of diversity was observed in the pericentromeric region of chromosome 19 and telomeric and sub-telomeric regions of chromosomes 2 and 1, respectively (**Figure S9**).

**Figure 9:**
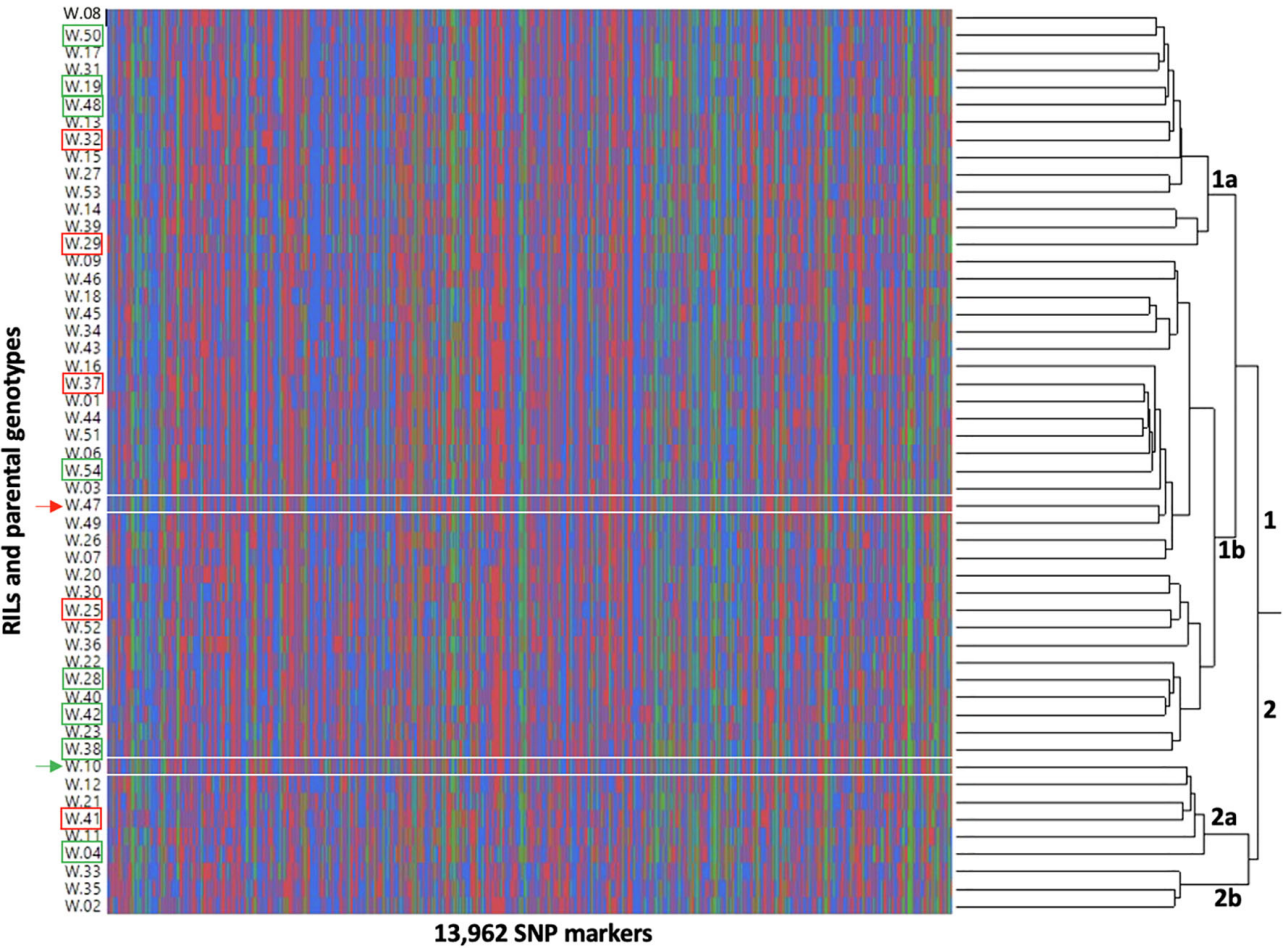
Dendrogram showing the interrelationship of 50 recombinant inbred lines (RILs) (W.01-W.54) and two parental genotypes [W.10 - ICGS76 (Heat tolerant) and W.47 - Tamrun OL02 (Heat susceptible)] based on the genotypic data. The dendrogram was produced using Ward’s hierarchical clustering method. The parental genotypes were marked by arrowheads (green depicting heat-tolerant genotype, ICGS76 and red depicting heat-susceptible genotype, Tamrun OL02). The most heat-tolerant and the most heat-susceptible RILs based on heat-adaptive lipid metabolism are marked by rectangles; green rectangles depict tolerance and red rectangles depict susceptibility.

Fifty RILs (excluding two RILs with low-depth sequences) and two parental genotypes were clustered based on the genotypic data (**Figure 9**). The analysis grouped genotypes into two major clusters, 1 and 2. Further, each cluster is sub-divided into two clusters, cluster 1 into 1a (14 RILs) and 1b (28 RILs and Tamrun OL02) and cluster 2 into 2a (5 RILs and ICGS76) and 2b (3 RILs). The best (green rectangles) and worst (red rectangles) RILs in terms of heat-adaptive lipid metabolism (decreases in 18:3 containing DGDG, MGDG, and PG and increases in 18:3 containing TG and SE) (clusters 4 and 6 on **Figure 8**) were additionally highlighted in **Figure 9**. Two parental genotypes clustered into separate clusters, and the most contrasting RILs in terms of the heat-adaptive lipid metabolism did not cluster together but instead grouped somewhat randomly.

**Figure 10:**
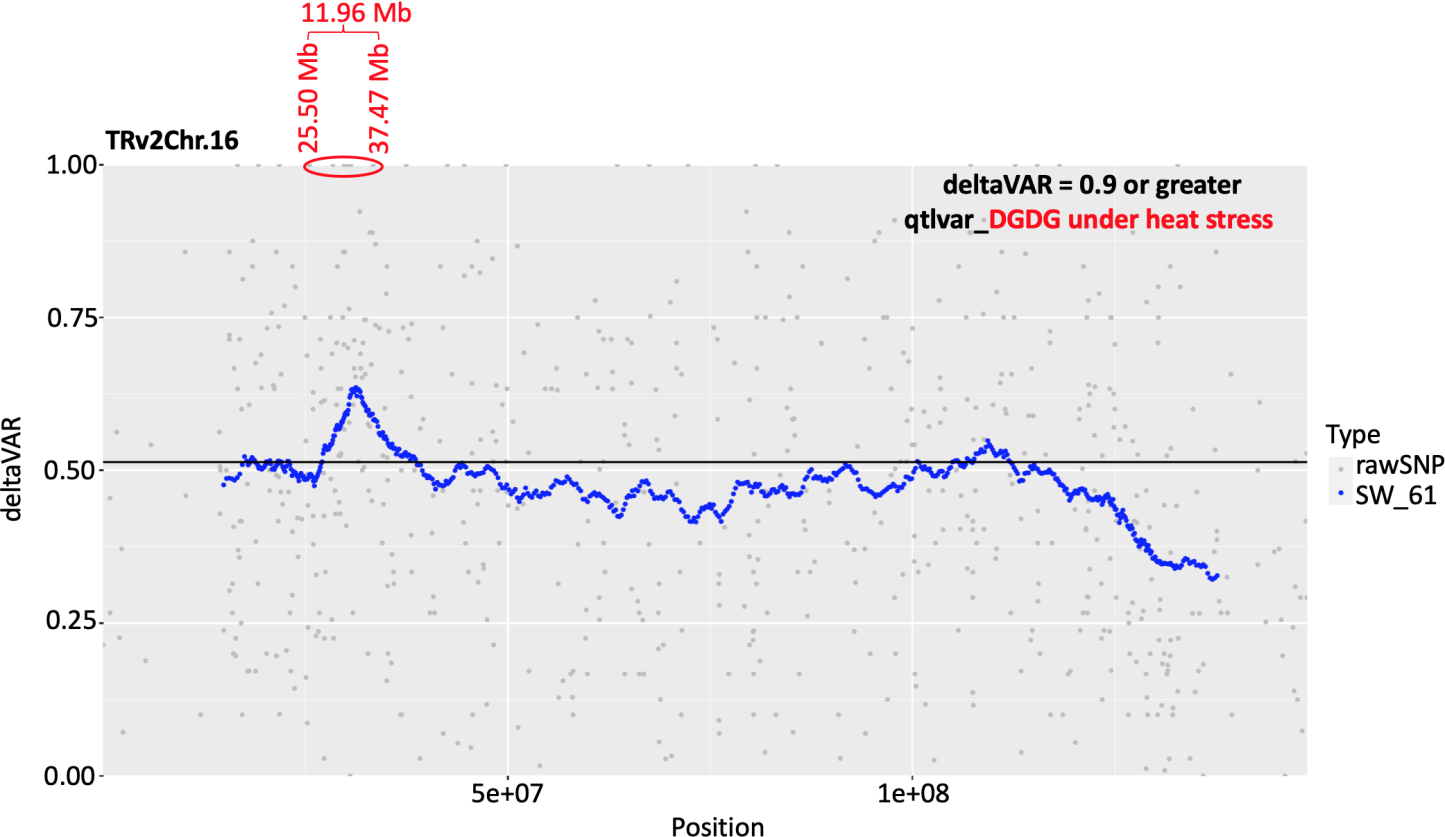
Plots showing conditional quantitative trait loci (QTL) detected for digalactosyldiacylglycerol (DGDG) lipids under heat stress on the peanut chromosome 16. This QTL spanned 11.98 Mb and 15 SNPs with a deltaVAR value of 0.9 or greater. The genomic coordinates for these SNPs are presented in **Supplementary Table S10**.

An association analysis was conducted using two physiological traits (chlorophyll index and the efficiency of PSII) and DGDG, MGDG, PG, TG, and SE species with the highest percent change between the temperature treatments (DGDG 18:3_18:3, DGDG 18:3_16:1, MGDG 18:3_16:1, MGDG 20:1_18:3, PG 18:3_16:1, PG 18:2_16:1, TG 18:3_18:3_18:3, TG 18:3_18:3_18:2, TG 18:2_18:3_18:2, and sitosterol 18:3). The RILs from the extreme tail of the distribution of a phenotypic trait (two physiological and ten lipid traits) were used for the association analysis which was conducted using the Khufu software package. Out of the 12 traits recorded under OT and HT, a signal was detected for DGDG lipids under HT on the peanut chromosome 16. This QTL spanned 11.98 Mb and 15 SNPs (**Figure 10**; **Table S10**). These markers will facilitate fine mapping of this QTL in the future.

## 4 Discussion

The decreases in chlorophyll index and the efficiency of photosystem II under heat stress demonstrate the heat stress impact on the physiology of the peanut plants (**Figure 1**). However, peanut plants still exhibited some cellular-level mechanisms that could be adaptive to heat stress. The remodeling in the leaf lipidome that occurred at high temperatures was in the expected direction to maintain membrane fluidity and minimize membrane damage (homeoviscous adaptation).

The major change in plastidic and extra-plastidic membrane lipids was the reduction in their unsaturation levels. The unsaturation index of DGDG, MGDG, PG, SQDG, PE, PC, and PI significantly decreased under heat stress (**Figure 3**). The unsaturated fatty acyl chains cause steric hindrance between membrane lipids, which in turn results in molecular disorder within the membrane bilayer (Los and Murata, 2004). Heating also reduces membrane compactness and increases membrane fluidity (Fan and Evans, 2015). By decreasing the number of double bonds in the fatty acid chains of membrane lipids under elevated temperatures, plants can enhance cell membrane compactness and maintain optimal membrane fluidity and integrity. Thus, in our study, the reduction in lipid unsaturation indices is consistent with the maintenance of cell membrane function under high temperatures in peanut.

We evaluated the species-level changes of DGDG, MGDG, PG, PC, PE, and PI, i.e., the lipid classes that composed 95% of the lipidome under OT and HT (**Figure 2A**). In all these lipid classes, species containing the 18:3 acyl chains decreased under HT (**Figures S5**, **S6**), resulting in a 27 to 47% decrease in 18:3 fatty acid levels under HT as compared to OT (**Table 1**). Along with that, the levels of all 18:3 acyl-containing TGs, except TG (52:6), i.e., TG 16:0_18:3_18:3, significantly increased under HT (**Figure 4A**). Together, these results indicate the sequestration of 18:3 acyl chains from membrane lipids into TGs to reduce the unsaturation levels of membrane lipids. In *Arabidopsis*, a soluble diacylglycerol acyltransferase (DGAT3) catalyzes the incorporation of 18:3 fatty acids into TGs in an acyl-CoA-dependent pathway of TG biosynthesis (Hernández et al., 2012). In an acyl-CoA-independent pathway, phospholipid:diacylglycerol acyltransferase (PDAT) catalyzes the transfer of an acyl group from the sn-2 position of a phospholipid, mainly PC, to the sn-3 position of DG, giving rise to TG and a lysophospholipid (Dahlqvist et al., 2000). Higashi et al. (2015) reported the overexpression of both PDAT and DGAT under heat stress. In our study, the total amount of TG increased under heat stress (**Figure 2C**), indicating a heat-induced accumulation of TG likely to remove the 18:3 acyl chains from membranes. Further, TGs clearly separated the temperature treatments into distinct clusters in a principal component analysis scores plot (**Figure 4B**) supporting the role of TGs in plant adaptation to heat stress. Our data are consistent with the TG pool acting as a sink for unsaturated fatty acids removed from membrane lipids in plants experiencing stress.

Sterol derivatives, i.e., SEs and SGs, also contributed to possible heat adaptation. In plants, SEs serve as a storage pool of sterols and are critical for membrane sterol homeostasis (Ferrer et al., 2017). In our study, the levels of the measured SEs, sitosterol 18:2 and sitosterol 18:3, significantly increased under heat stress (top two lipids in terms of percentage increase under HT). The 18:2 and 18:3 acyl chains increased by 5.4-fold and 2.5-fold, respectively, in SEs under HT (**Table 1**). Our data suggests the possibility of sequestration of unsaturated acyl chains (i.e., 18:2 and 18:3) from membrane lipids into sterols as SEs. This is supported by recent reports on the biosynthesis of SEs. Two sterol acyl transferases are currently characterized in plants, which are phospholipid:sterol acyl transferase (PSAT) and acyl CoA:sterol acyltransferase (ASAT) (Ferrer et al., 2017). The acyl donors for PSAT and ASAT are phospholipids and acyl CoA, respectively. It has been reported in *Caenorhabditis elegans* that acyl-CoA dehydrogenase drives heat adaptation by sequestering fatty acids from membrane lipids and thereby reducing substrates for FADs (Ma et al., 2015). This has been described as a mechanism to reduce the levels of unsaturated fatty acids in membranes and to reduce membrane fluidity. In our study, the 18:2 and 18:3 fatty acids that became esterified to sterols and formed SEs might have been transferred directly from phospholipids (PSAT mechanism) or from acyl-CoA (ASAT mechanism). Taken together, our results suggest that HT stress causes SEs to assume a novel role as buffers of polyunsaturated fatty acids from the membrane diacyl lipids.

Sterol glycosides function as membrane components, storage forms of sterols, transporters, and signaling molecules (Grille et al., 2010). Sterol glycosides have a condensing effect on membranes that helps to eliminate phase transitions to non-bilayer phases at high temperatures (Muramatsu et al., 2000). In a previous report on wheat, SGs also were found to increase upon exposure to HT, particularly when the wheat genotype was heat tolerant (Narayanan et al., 2016a). Taken together, the increase of 8-36% in SGs under HT in our study (**Figure 6**) is likely an adaptive response to HT.

The reduced level of 18:3 acyl-containing membrane diacyl lipids under HT can also be a result of reduced FAD activity. In the plant ER, FAD2 and FAD3 desaturate 18:1 and 18:2 acyl chains on PC. In the plastid, FAD5 desaturates 16:0 in MGDG, FAD6 desaturates 16:1 and 18:1 in MGDG and other plastidic lipid classes, and FAD7 and FAD8 desaturate 16:2 and 18:2 on MGDG and in other plastidic classes. Additionally, FAD4 converts 16:0 in PG to 3-*trans*-16:1. While it’s difficult to guess FAD6 activity from the steady-state levels of substrates and products, the patterns of molecular species observed suggest decreases in the function of the other FADs: i.e., the buildup of PC 16:0_18:1 and a decrease in PC 16:0_18:3 suggest decreases in FAD2 and FAD3 activity, the buildup of PG 16:0_16:0 and decrease in PG 16:0_16:1 is consistent with a decrease in FAD4 activity, the increase in MGDG 18:3_16:0 and decrease in MGDG 18:3_16:1 with a decrease in FAD5 activity, and the increase in MGDG 18:2_18:3 and decrease in MGDG 18:3_18:3 is consistent with a decrease in FAD7/8 activity. Along the same lines, in our previous research, we observed that several peanut varieties decreased lipid unsaturation levels by lowering 18:3 fatty-acid levels through reduced *FAD3* gene expression (Zoong Lwe et al., 2020).

A reduction in FAD3, FAD7, and FAD8 activities could lead to an accumulation of 18:2 fatty acids. However, in our study, 18:2 fatty acid levels exhibited a fold decrease under HT in most diacyl lipids (**Table 1**). This was accompanied by significant fold increases of 18:2 acyl chains in TG and SE (**Table 1**). Taken together, our results indicate that the peanut plants might have minimized the increases in 18:2 acyl chains in membrane diacyl lipids by possible sequestration of 18:2, along with 18:3, in TGs and SEs.

The results from this study provide evidence for the regulation of fatty acid oxidation in plants to adapt to heat stress. Under heat stress conditions, the oxidative burst resulting from the accumulation of ROS can increase the rate of reaction of PUFAs with ROS leading to sequential lipid peroxidation reactions (Vistoli et al., 2013). As a result, lipids may be degraded, and the fluidity and integrity of membranes may be compromised (de Dios Alché, 2019). Lipid peroxidation and the generation of harmful lipid-oxidation products depend upon the degree of fatty acid unsaturation (Mano et al., 2014). This was evident in *fad7fad8 Arabidopsis* mutants (FAD7/FAD8 converts 18:2/16:2 acyl chains to 18:3/16:3 in the chloroplast) that were defective in fatty acid desaturases and showed a lower level of lipid-oxidation products (Mano et al., 2014). This could likely be due to the decreased availability of 18:3 or 16:3 acyl chains to scavenge reactive oxygen species (i.e., reduced level of fatty acid precursors). Additionally, it has been reported that fatty acid desaturases may have intrinsic hydroxylase activity due to the fundamental mechanistic similarities between desaturation and hydroxylation (Broadwater et al., 2002). Thus, mutants with lower levels of desaturase activity may also demonstrate a lower level of oxidized lipids. In our study, a reduction in 18:3 acyl chains in membrane lipids under heat stress might have helped to reduce the availability of PUFAs for oxidation, which in turn limits lipid peroxidation and damage to membranes. For this mechanism to be integrated into the overall whole-plant level heat tolerance mechanisms, some other components of the antioxidant system (e.g., antioxidant enzymes) might also need to be efficient to reduce oxidative damage.

The interplay among the remodeling of membrane diacyl lipids containing 18:3 acyl chains, TGs, SEs, and lipids with oxidized 18:3 acyl chains was evident through the analysis of lipid co-occurrence and coordinated metabolism (**Figure 7**). The coordinated metabolism of membrane diacyl lipids with 18:3 acyl chains grouped them into a single cluster on a dendrogram (cluster 1; **Figure 7**). Similarly, the coordinated metabolism of TGs, SEs (sitosterol 18:2 and sitosterol 18:3), and lipids with oxidized 18:3 acyl chains was evident from their clustering (TGs, and SEs in cluster 4 and ox-lipids in cluster 1, 2, and 3). The negative correlation between cluster 1 and cluster 4 supports possible interconversions; i.e., sequestering of 18:3 acyl chains from membrane diacyl lipids into TGs and SEs. Similarly, the positive correlation of cluster 1 with clusters 2 and 3 supports the coordinated downregulation of lipids with oxidized 18:3 acyl chains and the 18:3 acyl-containing membrane diacyl lipids.

**Figure 11:**
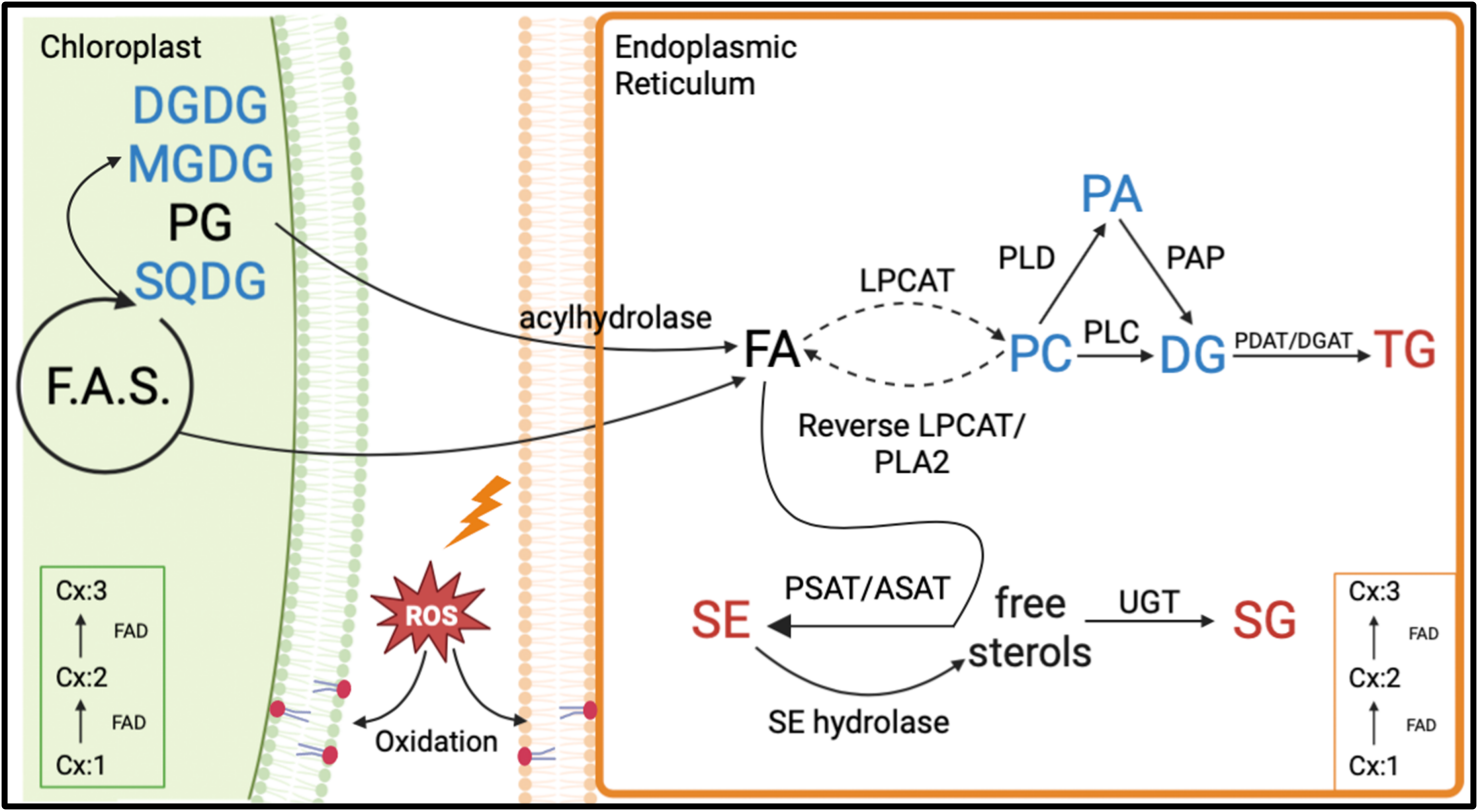
Proposed lipid biochemical pathways involved in lipid remodeling under heat stress in peanut. Lipid classes in red font color denote significant increases (α=0.05) in amounts under heat stress. Lipid classes in blue font color indicate significant decreases (α=0.05) in amounts under heat stress. Lipid classes in black font color experienced no significant (α=0.05) changes under heat stress. Dashed arrows indicate multi-step conversions. Cx:1 to Cx:3 indicates fatty acids with a specific number of carbon atoms: double bonds. The figure is based on the general route of plant lipid biosynthesis in which fatty acids (FA) synthesized in the plastid are incorporated into plastidic lipids or exported to the endoplasmic reticulum (ER) and incorporated into extra-plastidic lipids with or without further modifications. Fatty acids undergo desaturation through the action of fatty acid desaturases (FADs) in the chloroplast and ER. Fatty acids can be hydrolyzed from the plastidic and extra-plastidic lipids by various acyl hydrolases (Grienenberger et al., 2010; Higashi et al., 2015). Fatty acids can be released from phosphatidylcholine (PC) via the reverse reaction of lysophosphatidylcholine acyl transferase (LPCAT) or via phospholipase A2 (PLA2). PC can be converted to diacylglycerols (DG) by removal of the entire head group via phospholipase C (PLC), the removal of the PC headgroup by phospholipase D (PLD) producing phosphatidic acid (PA) followed by removal of the remaining phosphate by phosphatidic acid phosphatase (PAP) (Zhang et al., 2017; Shiva et al., 2020). DG can be incorporated into triacylglycerol (TG) along with fatty acids via phospholipid:diacylglycerol acyltransferase (PDAT) or diacylglycerol acyltransferase (DGAT) (Dahlqvist et al., 2000; Schnurr et al., 2002; Hernández et al., 2012). Fatty acids can be incorporated into phytosterols by sterol acyl transferases such as phospholipid:sterol acyltransferase (PSAT) and acyl-CoA:sterol acyltransferase (ASAT), forming sterol esters (SEs) (Ferrer et al., 2017). A reverse reaction that involves the hydrolysis of SEs can release fatty acids from SEs, forming free sterols (Shiva et al., 2020). Sterols can be converted to sterol glycoside (SGs) by the action of UDP-glucose transferase (UGT) enzymes that transfer glucose from a UDP-glucose to a sterol (Schrick et al., 2012; DeBolt et al., 2009). Fatty acids incorporated in membrane diacyl lipids can be oxidized through the action of multiple enzymes such as a lipoxygenase, allene oxide synthase, allene oxide cyclase, and oxophytodienic acid reductase or through the reaction with singlet oxygen and radical chain oxidation (de Dios Alché, 2019). Other abbreviations used in the figure: DGDG, digalactosyldiacylglycerol; F.A.S., fatty acid synthesis; MGDG, monogalactosyldiacylglycerol; PG, phosphatidylglycerol; ROS, reactive oxygen species; SQDG, sulfoquinovosyldiacylglycerol; TrGDG, trigalactosyldiacylglycerol.

A lipid biochemical pathway map places the observed lipid remodeling within the broader context of plant lipid metabolism (**Figure 11**). As depicted in the figure, the loss of fatty acids, particularly 18:3 from plastidic and non-plastidic lipids, with concomitant increases in SE and TG, suggests the movement of fatty acids through a fatty acid pool into the neutral lipid buffers. This process is likely to involve the activation of hydrolytic activities, transport of fatty acids out of the plastid, and enzymes catalyzing re-esterification of the fatty acids. A hierarchical clustering grouped the RILs that demonstrated the heat-adaptive lipid metabolism (decreases in 18:3 containing DGDG, MGDG, and PG and increases in 18:3 containing TG and SE) at the greatest extent with the heat-tolerant parent, ICGS76, and the RILs in the opposite extreme with the heat-susceptible parent, Tamrun OL02 (**Figure 8**), further supporting the involvement of lipid remodeling in heat tolerance. Finally, our study linked the observed phenotypic changes to the genotype (**Figures 9**, **10**) and generated information that would be useful for identifying molecular markers for heat tolerance.

A more-than-expected number of SNPs were detected on four A sub-genome chromosomes (1, 2, 4, and 6) and two B sub-genome chromosomes (16 and 19) (**Figure S8**). The expected number of markers per chromosome and sub-chromosomal regions were calculated based on chromosome sizes. It is apparent from this analysis that markers are non-randomly distributed in the peanut genome, subgenomes, chromosomes, and sub-chromosomal regions. Indeed, according to earlier research, the sub-telomeric chromosomal regions exhibit highly polymorphic levels due to high recombination frequency (Aguilar and Prieto, 2021), which testifies to a biased distribution of markers in plant genomes. Much like Bertioli et al. (2019), this study observed more polymorphism in B relative to the A sub-genome of peanut. Further, in this study, a hike in polymorphism is observed in chromosome 19. A literature search suggested the presence of a large inversion in chromosome 19 of the reference genotype ‘Tifrunner’ (Bertioli et al., 2019). Since the reference genome was used to align the sequences in this study, likely, the parental genotypes, Tamrun OL02 (heat susceptible) and ICGS76 (heat tolerant), of this population do not carry similar inversion, leading to this jump in the level of polymorphism. Clustering of the genotypes from the two extremes of the phenotypic distribution of the lipid traits was not expected as these genotypes might not carry similarity at the genome-scale but may share similarity at a specific chromosome region contributing to this trait.

Mapping of a QTL for the DGDG production under heat stress represents an inducible regulatory gene that determines the production of these plastidic lipids under heat stress and may serve as a marker to select for this biochemical trait (**Figure 10**). Fifteen markers underlie this QTL, which will serve as a valuable resource during fine mapping for this locus. In the future study, the candidate genes underlying this 11.96 Mb interval will be carefully examined for their functions and classified based on annotations, and the ones that look promising will be functionally characterized using gene silencing and functional complementation. These regulatory genes, once identified, could serve as genetic markers to select for heat tolerance in peanut. It might be that the abundance of DGDG in the leaf lipidome (30% in the current study) corroborates the heavy fatty acid translocation flux between DGDG and TAGs or SEs, thus making DGDG important for stress responses.

## 5 Conclusions

This study, which examined a peanut population of 52 RILs plus the parental genotypes, elucidates lipid remodeling in peanut under heat stress. The major adaptation mechanism in the peanut leaf lipidome under HT is the reduction in the unsaturation levels of plastidic and extra-plastidic membrane diacyl lipids. A reduction in unsaturation is achieved predominantly through the reduction in the 18:3 acyl chains. In our study, the levels of 18:3-containing triacylglycerols increased under HT, consistent with their possible role in sequestering fatty acids during membrane lipid remodeling via the acyl-chain remodeling mechanism. Our study also found a novel mechanism of acyl chain recycling under HT, i.e., by sequestering the polyunsaturated acyl chains from membrane diacyl lipids into sterols, forming SEs. The sequestration of 18:3 acyl chains from the membrane diacyl lipids also appears to be a mechanism for decreasing the availability of susceptible molecules for oxidation. Taken together, our results suggest that the interplay among the 18:3-acyl-containing membrane diacyl lipids (downregulation under HT), 18:3-acyl-containing TGs (upregulation under HT), and polyunsaturated acyl-containing SEs (upregulation under HT) is at the core of lipid remodeling for heat stress adaptation in peanut. We speculate that lipid remodeling may prevent the phase transition of membranes from a liquid crystalline (bilayer) phase to a non-bilayer phase at high temperatures and maintain optimal membrane fluidity, integrity, and function. Our study also linked the observed heat-adaptive lipid remodeling to the genotype and identified a genomic region associated with heat-adaptive lipid remodeling, which would be useful for identifying molecular markers for heat tolerance that will aid in breeding heat-tolerant peanut varieties.

## Data availability statement

The datasets presented in this study can be found in online repositories. The names of the repository/repositories and accession number(s) can be found in the article/**Supplementary Material**.

## Author contributions

WS: Data curation, Formal analysis, Investigation, Methodology, Software, Validation, Visualization, Writing – original draft, Writing – review & editing. SR: Data curation, Methodology, Supervision, Validation, Writing – original draft, Writing – review & editing. RW: Data curation, Resources, Writing – review & editing. MR: Data curation, Formal analysis, Writing – review & editing. MB: Resources, Writing – review & editing. WB: Formal analysis, Resources, Supervision, Validation, Writing – review & editing. SN: Conceptualization, Formal analysis, Funding acquisition, Methodology, Project administration, Resources, Supervision, Validation, Writing – review & editing.
