# Supplementary material for: Lipid modulation contributes to heat stress adaptation in peanut
Source: Front Plant Sci. 2023 Dec 18;14:1299371. doi: 10.3389/fpls.2023.1299371 (PMC10757947; doi:10.3389/fpls.2023.1299371)
Supplement: Supplementary file 1 [file DataSheet_1.docx]

Supplementary Material

**Supplementary Figure S1.** The observed air temperature inside the greenhouse (a) and growth chamber (b) during a representative 24 h period. Plants were exposed to optimal temperature (OT) in the greenhouse and high temperature (HT) inside the growth chamber. The experiment was conducted twice, as indicated by the blue and red traces.


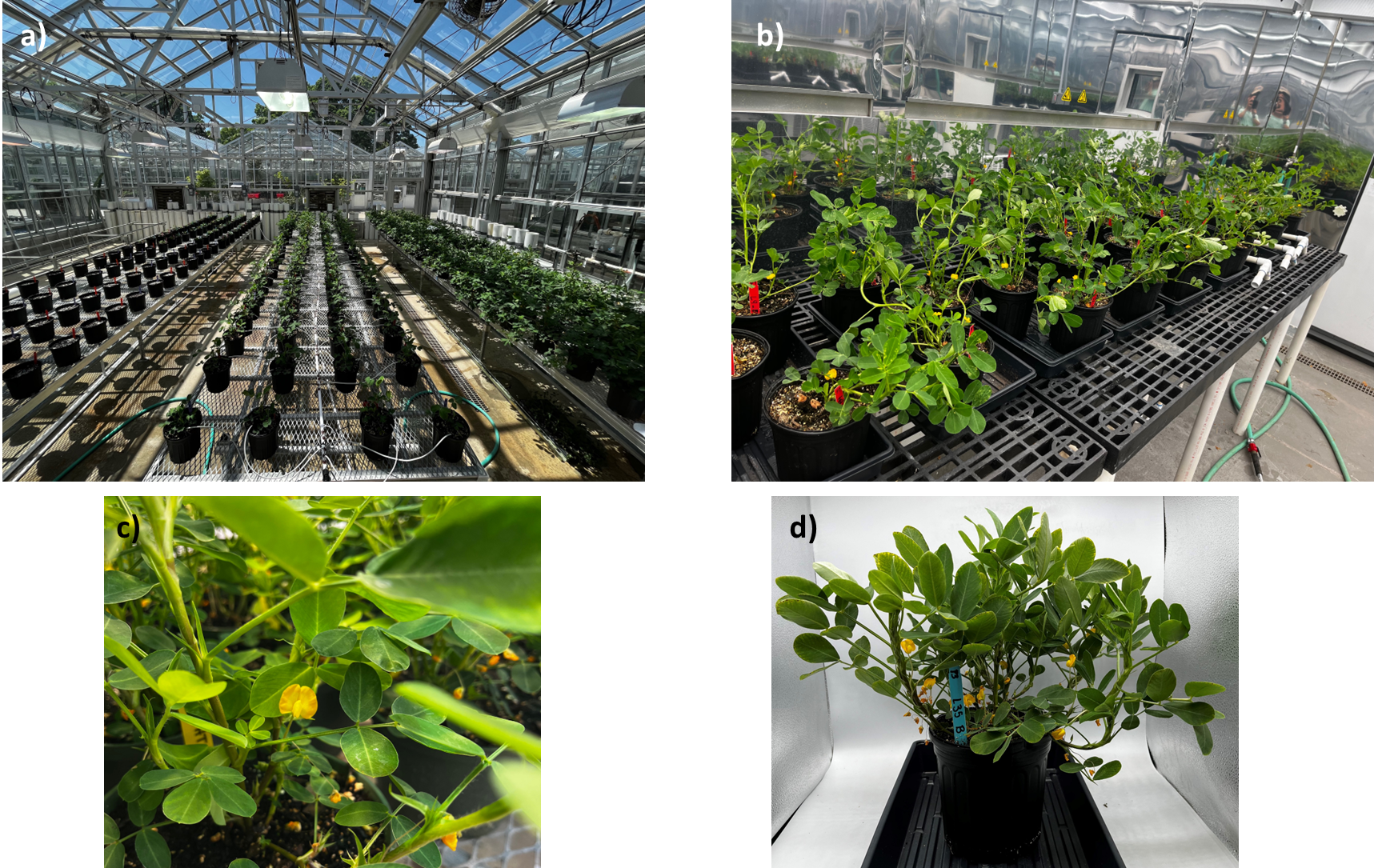


**Supplementary Figure S2.** Plants growing in the greenhouse (optimal temperature) (a) and growth chamber (high temperature) (b). A closer view of a plant growing in the greenhouse and growth chamber is given in images ‘c’ and ‘d’, respectively.


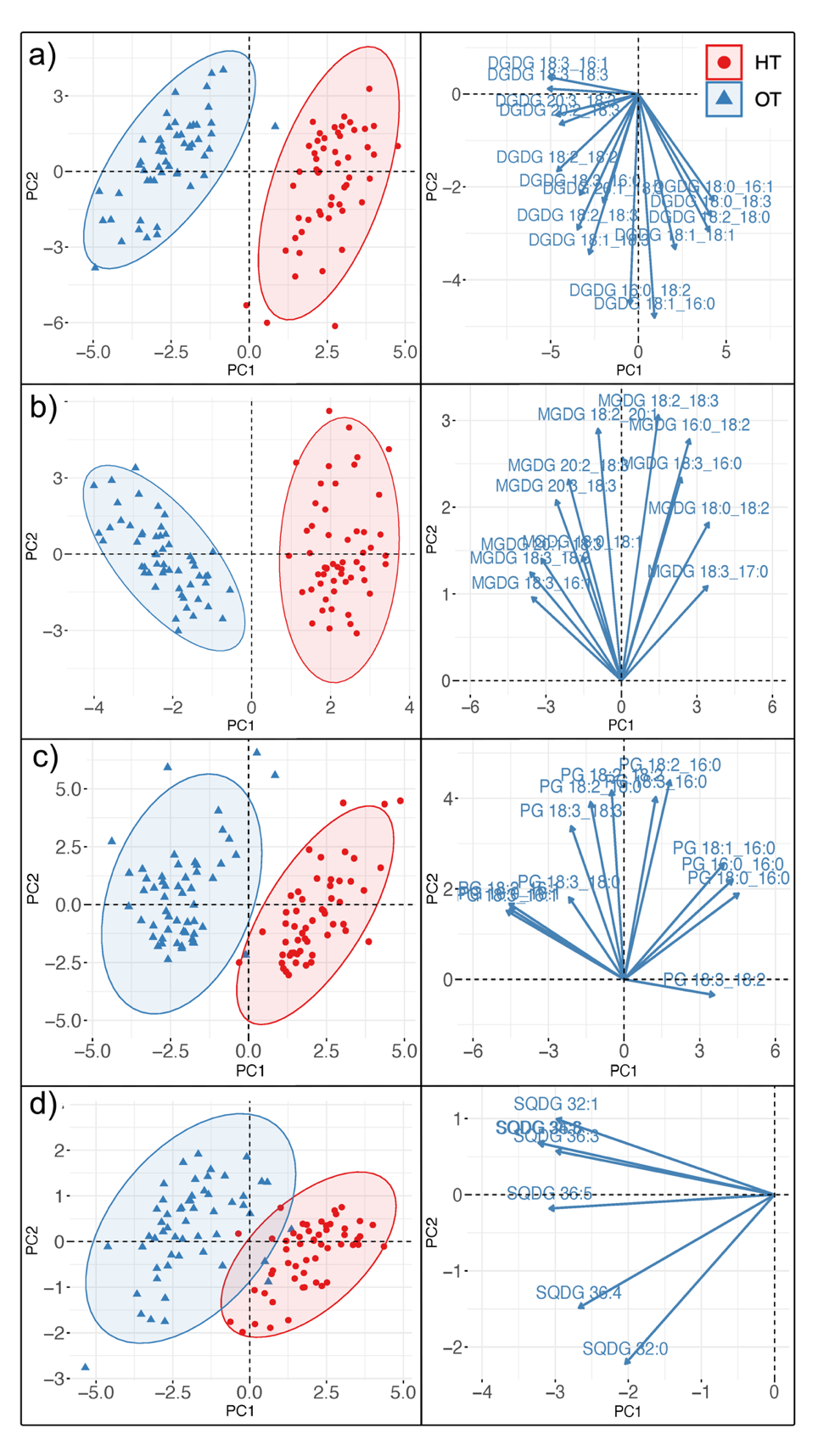


**Supplementary Figure S3.** Principal Component Analysis (PCA) biplots demonstrating the differentiation of the two treatments: optimal temperature (OT, 30/20°C) and high temperature (HT, 38/28°C) by various molecular species of plastidic lipids, digalactosyldiacylglycerol (DGDG) (a); monogalactosyldiacylglycerol (MGDG) (b); phosphatidylglycerol (PG) (c); and sulfoquinovosyldiacylglycerol (SQDG) (d). Lipid molecular species are identified as total acyl carbons:total double bonds.

_
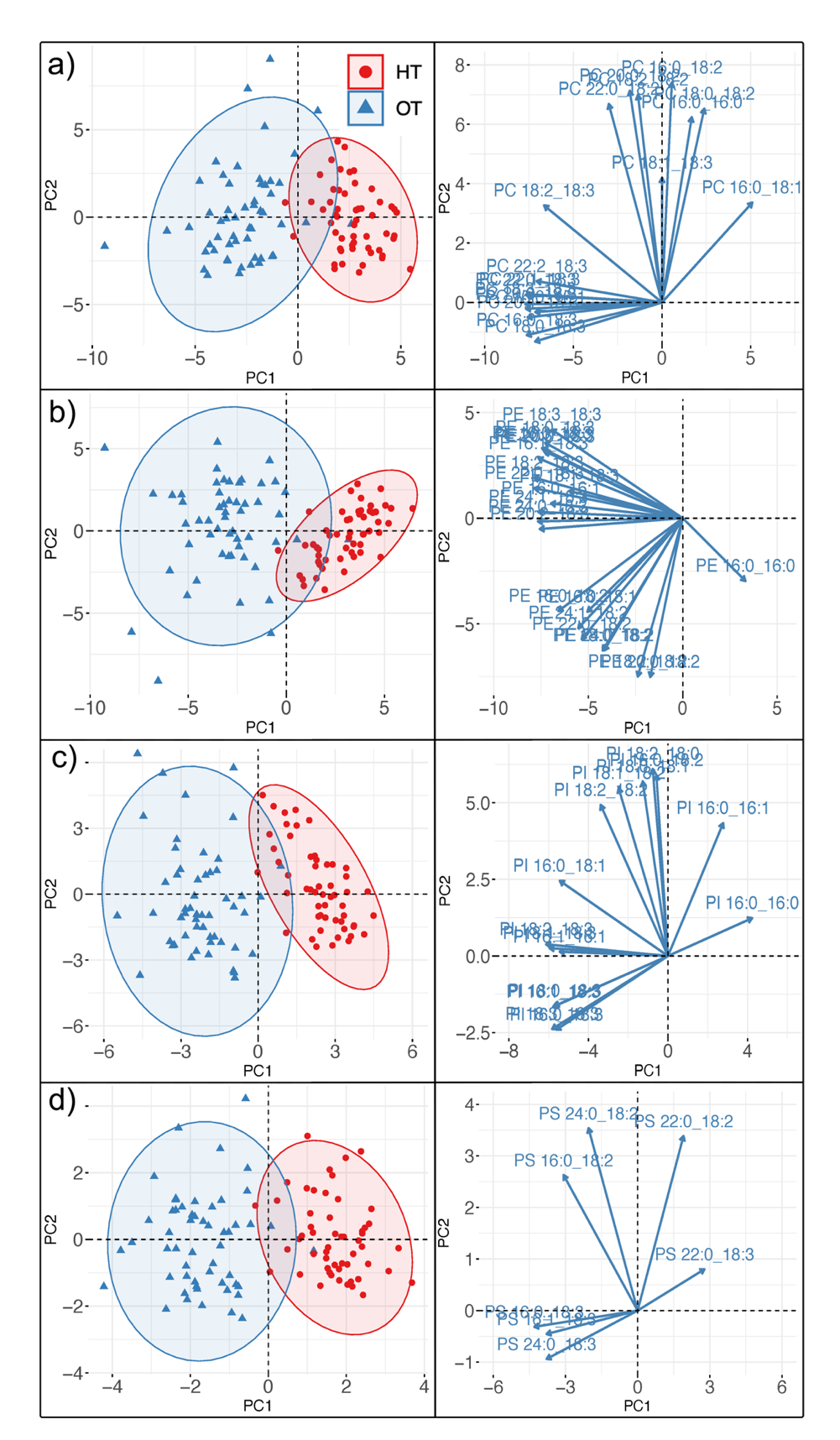
_

**Supplementary Figure S4.** Principal Component Analysis (PCA) biplots demonstrating the differentiation of the two treatments: optimal temperature (OT, 30/20°C) and high temperature (HT, 38/28°C) by various molecular species of extra-plastidic lipids, phosphatidylcholine (PC) (a); phosphatidylethanolamine (PE) (b); phosphatidylinositol (PI) (c); and phosphatidylserine (PS) (d). Lipid molecular species are identified as total acyl carbons:total double bonds.


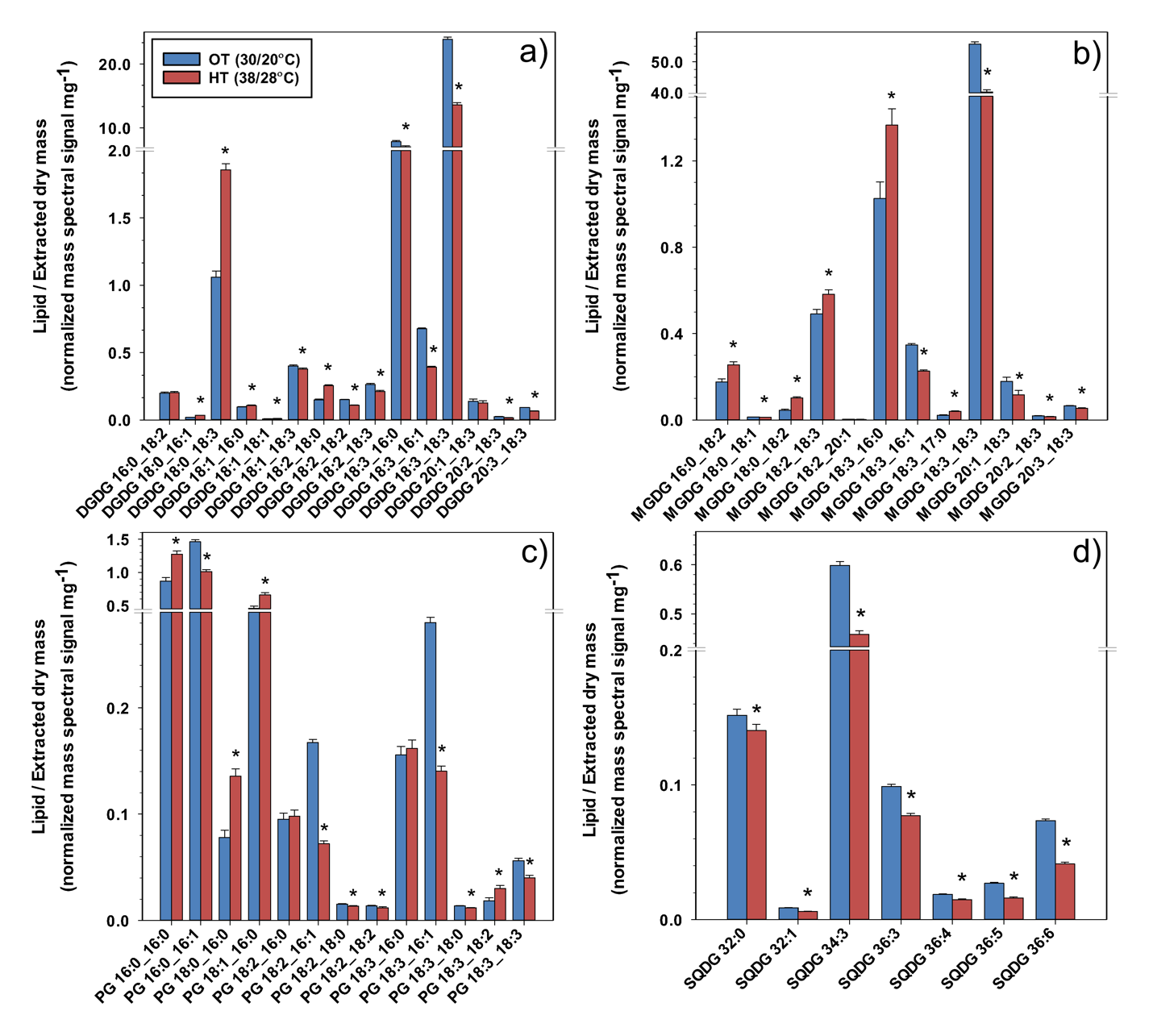


**Supplementary Figure S5.** Temperature effects on the molecular species of digalactosyldiacylglycerol (DGDG) (a), monogalactosyldiacylglycerol (MGDG) (b), phosphatidylglycerol (PG) (c), and sulfoquinovosyldiacylglycerol (SQDG) (d). The values shown are the least-squares means. Error bars represent the standard errors of the least-squares means of 324 observations (2 experimental runs x 54 genotypes x 3 replications). A break on the y-axis indicates a change in scale. An asterisk (*) above the bars indicates a significant difference between optimum temperature (OT) and high temperature (HT) at α = 0.05 according to the Fisher’s least significant difference (LSD) test.


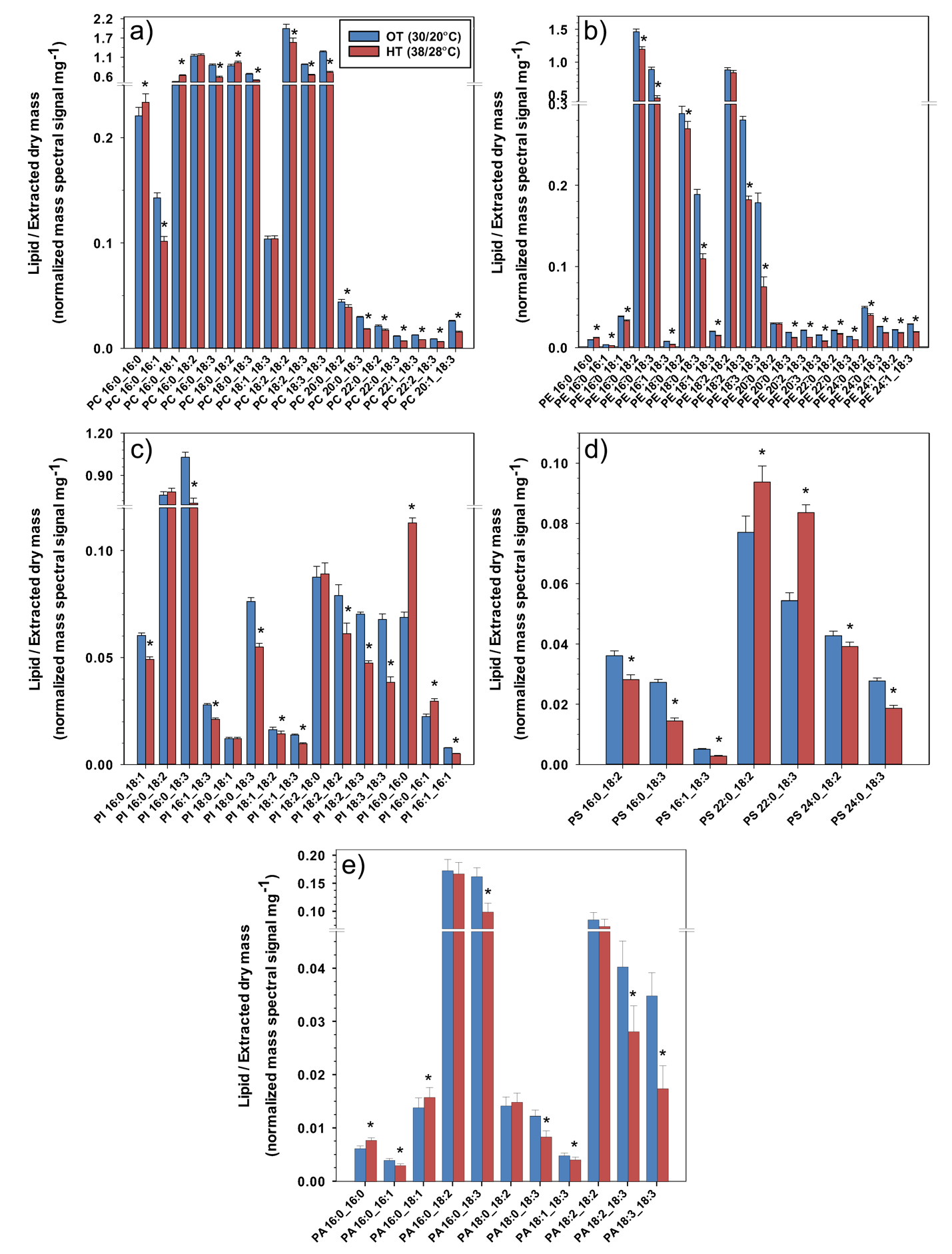


**Supplementary Figure S6.** Temperature effects on the molecular species of phosphatidylcholine (PC) (a), phosphatidylethanolamine (PE) (b), phosphatidylinositol (PI) (c), phosphatidylserine (PS) (d), and phosphatidic acid (PA) (e). The values shown are the least-squares means. Error bars represent the standard errors of the least-squares means of 324 observations (2 experimental runs x 54 genotypes x 3 replications). A break on the y-axis indicates a change in scale. An asterisk (*) above the bars indicates a significant difference between optimum temperature (OT) and high temperature (HT) at α = 0.05 according to the Fisher’s least significant difference (LSD) test.


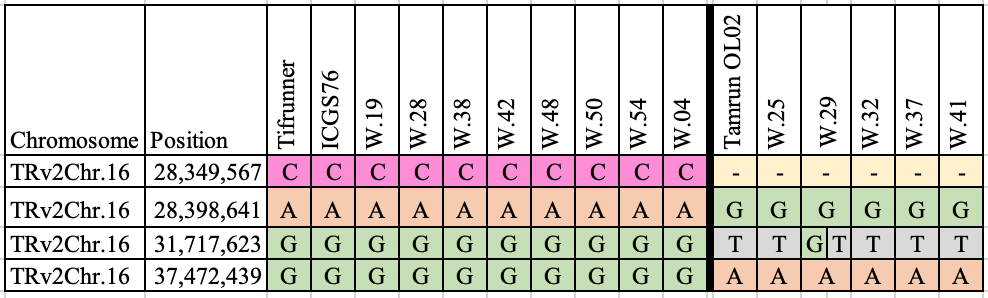


**Supplementary Figure S7.** The SNP haplotype of the genotypes in cluster 3 and cluster 6 of Figure 8 along with that of the reference peanut genotype Tifrunner used for DNA sequencing (Bertioli et al., 2019). The cluster 3 of Figure 8 included the heat-tolerant parental genotype, ICGS76 (W.10) and the RILs showing heat-adaptive lipid metabolism (W.19, 28, 38, 42, 48, 50, 54, and 4), whereas cluster 6 included the heat-susceptible parental genotype, Tamrun OL02 (W.47) and the RILs that exhibited unfavorable heat-responsive lipid metabolism (W.25, 29, 32, 37, and 41).


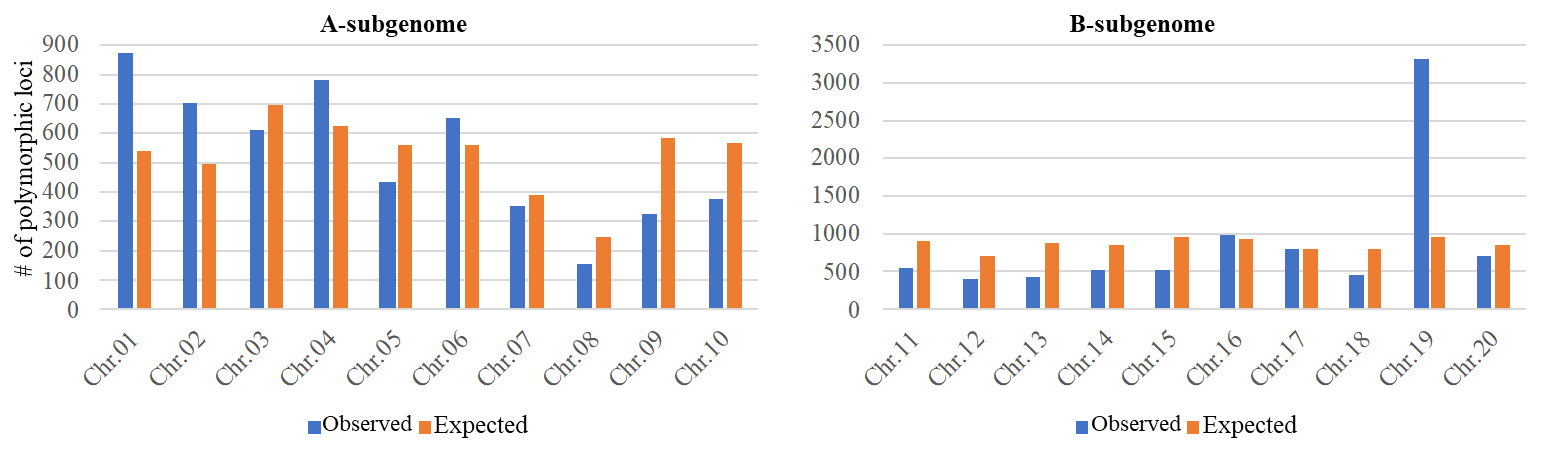


**Supplementary Figure S8.** Plots showing the distribution of SNP markers in the peanut sub-genomes. The observed number of markers was compared with the expected number of markers determined based on peanut chromosome sizes adapted from Bertioli et al. (2019) (see material and methods for further details). We calculated marker density per Mb for peanut sub-genome A and B, 4.83 markers/Mb and 6.01 markers/Mb, respectively, based on the total number of SNPs observed in the present study and the chromosome and sub-genome sizes determined in Bertioli et al. (2019). These numbers were used to calculate the expected number of markers per chromosome, given a random (unbiased) distribution of molecular markers. When compared with the observed number of SNPs, a more-than-expected number of SNPs were observed for chromosomes 1 (331), 2 (206), 4 (156), and 6 (96) of A-subgenome and chromosomes 16 (50) and 19 (2347) of the B-subgenome. Since many other chromosomes exhibited less than expected number of SNPs, which range from 16 to 448 for A and B subgenomes, these changes were not treated as structural changes in peanut chromosomes but were considered genotypic differences, and only a burst in the observed number of SNPs observed for chromosome 19 (2347; observed 3300 and expected 812) was treated as a structural change.


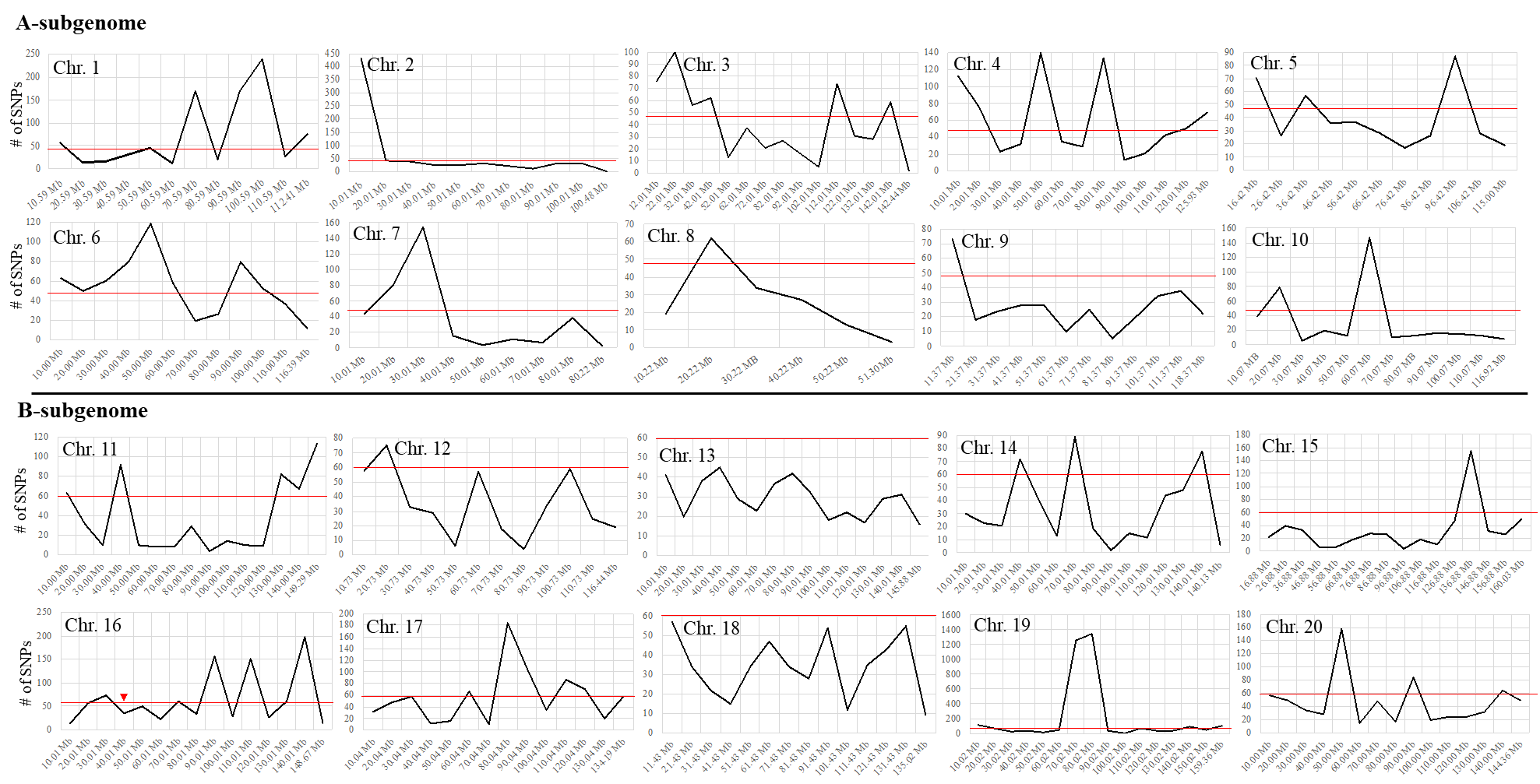


**Supplementary Figure S9.** Plots showing the distribution of SNP markers at the sub-chromosomal level. Each chromosome was divided into 10 Mb bins for ease of presentation, and the number of markers mapping in each bin was plotted. The observed number of markers was compared with the expected number of markers determined based on peanut chromosome sizes adapted from Bertioli et al. (2019) (see material and methods for further details). The expected number of markers was highlighted by a red line.

**Data Availability:**

The genotype data for this publication can be publicly accessed in the PeanutBase Gigwa tool at <https://www.peanutbase.org/gigwa/>
